# Antennal Transcriptome of the Fruit-Sucking Moth *Eudocima materna*: Identification of Olfactory Genes and Preliminary Evidence for RNA-Editing Events in Odorant Receptors

**DOI:** 10.3390/genes13071207

**Published:** 2022-07-06

**Authors:** Meenal Vyas, Kamala Jayanthi Pagadala Damodaram, Gandham Krishnarao

**Affiliations:** Division of Crop Protection, ICAR-Indian Institute of Horticultural Research (IIHR), Hessaraghatta Lake Post, Bengaluru 560089, Karnataka, India; meenalvyas77@gmail.com (M.V.); gandhamkrishna42@gmail.com (G.K.)

**Keywords:** fruit-sucking moth, odorant-binding proteins, RNA editing, antennal transcriptomics, pheromone-binding proteins

## Abstract

Unappealing shriveled fruits are a characteristic of one of the most elusive fruit pests. The perpetrator, *Eudocima materna*, attacks the fruit at a fully formed stage and, therefore, the antennal transcriptome for this insect was deduced to identify the molecular elicitors involved in the attraction to its host plants. A total of 260 olfactory genes, including 16 odorant-binding proteins (OBPs), four pheromone-binding proteins (PBPs), 40 antennal-binding proteins (ABPs), 178 odorant receptors (ORs), 17 chemosensory proteins (CSPs) and five sensory neuron membrane proteins (SNMPs) were identified. Phylogenetic analysis shows the divergence of *E. materna* proteins from closely related lepidopterans and provides insights on genes that have exclusively evolved in this insect. STRING network analysis revealed interactions of olfactory proteins among themselves and the proteins of other groups. Interestingly, online tools predicted RNA-editing events in the odorant receptor sequences, suggesting the possibility of multiple protein forms. Transcripts matching transposable element sequences were also detected in the dataset. Thus, the work reported here provides a valuable resource to design molecular methods for pest control.

## 1. Introduction

Most lepidopterans (moths and butterflies) are agricultural pests that only damage the crop in the larval stage, while the adults are usually short-lived and harmless, surviving on nectar/plant juices until they mate and lay eggs. Among the handful of moths that are actually damaging in the adult stage, the fruit-sucking moth (FSM), *Eudocima* (*Othreis*) *materna* L. stands out. Its ability to attack several commercial fruit crops in tropical and subtropical regions from Africa to the Pacific Islands results in huge economic losses across the globe [1,2,3,4]. Male and female adult moths of *E. materna* and related species such as *Eudocima phalonia*, *Eudocima salaminia*, and *Eudocima homaena* are nocturnal feeders on fruits and cause extensive damage [1,2,3]. The moths attack fully developed fruits by piercing through the epicarp and sucking the juice, hence being commonly known as fruit-piercing or fruit-sucking moths [4]. The rotting of infested fruits is the collateral damage caused by secondary infestations by pathogens [5]. The female moths lay eggs on Menispermaceae plants such as *Tinospora cordifolia* that are abundant in forest and scrublands far away from the commercial fruit orchards [2], and the larvae feed on the foliage and develop into adults. Traditional management methods such as bagging, the hand-collection of moths, netting the orchards, night watching, insecticidal application, and bonfires to attract and kill the moths are of limited value in containing the fruit damage, as adult moths can fly long distances [6]. Therefore, the behavior-modifying strategies involving chemical cues such as sex pheromones and host cues (kairomones) that target insect olfaction/chemoreception are of immense value, as these are eco-friendly and sustainable [6].

First described by Carl Linnaeus in 1767, the elusive nature of the FSM has kept its chemo-ecological behavior relatively unexplored. Specialized aspects of *E. materna*, such as the survival of adults exclusively on high-acid content, sugar-rich fruit juices and the ability of the larvae to feed on the Menispermaceae family of plants that have insecticidal properties [7] make them increasingly worthy of investigation. Therefore, exploring the specific antennal olfactory proteins that aid the moths while foraging for feeding and oviposition sites is imperative. Deciphering the molecular basis of olfaction and odor perception with regard to their host/mate location and oviposition site selection will help in formulating the most effective behavior-modifying management strategies.

Antennae are an important asset to an insect’s olfactory system, facilitating chemoreception that detects cues for food location, mate identification, oviposition site selection, and threat detection [8,9]. The antennae bear sensilla [10] with a sensillum lymph consisting of olfactory proteins that can bind to the hydrophobic volatile cues in the environment [11]. Insects have evolved certain olfactory proteins such as chemosensory proteins (CSPs) and odorant-binding proteins (OBPs) that bind to the volatile cues from the environment and carry them further for recognition by the chemosensory neurons, which are detected by various olfactory receptors (ORs), gustatory receptors (GRs), or ionotropic receptors (IRs) [12,13,14,15,16,17,18]. Lepidopterans also have a few specialized OBPs, commonly referred to as PBPs, that detect pheromones [19].

Recent transcriptome (whole-body and antennal) studies have led to the identification of several OBPs and ORs across the insect community. However, transcriptome data of lepidopteran pests such as *Chilo supressalis* (Walker) [20], *Conogethes punctiferalis* (Guenée) [21], *Plodia interpunctella* (Hübner) [22], *Spodoptera exigua* (Hübner) [23], *Cnaphalocricis medinalis* (Guenée) [24,25,26], and *Plutella xylostella* (Linn.) [27] are insufficient to explore the olfactory genes of FSMs that are distinct from other lepidopterans in their behavior, as explained earlier. Significant details of a de novo antennal transcriptome were reported recently in another fruit-piercing noctuid moth, *Oraesiae marginata* Fab. The larvae of this moth develop on the plants of Menispermaceae and the adults are pests of several commercial fruit crops [28], much like the *E. materna* larval stages. Despite the similarity, there could be specifics that are not common to the two FSMs mentioned and several others that have not been discussed. Interestingly, a study comparing the OBPs from 12 *Drosophila* spp. genomes has shown that species that are specialized with regard to the ecological system have rapid evolution in the OBPs [29].

The behavioral chemical ecology requires the involvement of several receptors that are processed in the brain [16]. Although RNA-editing events are a well-known phenomenon that brings about protein diversity from a single gene, its importance in insects has been discovered in the last decade. RNA editing has been identified as a mechanism that ensures proper functioning of the brain in some model systems [30,31,32]. Transcript-specific modifications are brought about by the RNA-editing molecular mechanism, resulting in protein diversity [30,31,32]. The adenosine-to-inosine RNA editing mediated by adenosine deaminases acting on RNA or ADARs [33,34] is one such mechanism reported across several eukaryotes. Studies in different systems have shown that significant RNA editing exists in the brain [31]. More recently, significant RNA-editing events have been reported in bumblebees that manifest into specialized tasks [35]. Consequently, it would be interesting to know whether such RNA-editing events are a part of the machinery in insect pests that helps them adapt to a larger host range. Significant findings such as the one stated above underscore the importance of elucidating the olfactory genes of *E. materna* through antennal transcriptomics, which will eventually help comprehend its olfactory mechanism in detail and provide details on its commonalities with other fruit-sucking/piercing moths.

In the present study, the antennal transcriptome of *E. materna* was obtained and assembled to identify the various genes involved in its olfaction/chemosensation. Additionally, the phylogenetic analysis of the identified gene classes highlights the relationship of FSM with other closely related lepidopterans. STRING analysis using online tools helped to identify interactions between the different olfactory genes. Preliminary bio-informatic studies were also carried out to identify RNA-editing sites in some categories of the olfactory proteins that would help gain insights into how they govern insect behavior.

## 2. Materials and Methods

### 2.1. Insects

The insects were collected during larval development directly from the *Tinospora cardifolia* blocks of ICAR-IIHR, Bengaluru, Karnataka. The larvae were reared in the laboratory on natural *T. cardifolia* cuttings to complete development under controlled conditions (26 ± 1 °C, 75 to 80% RH). The emerged adults (one pair) were placed in oviposition cages (30 × 45 cm) lined with paper towels as oviposition substrate and covered with nylon mesh on the upper side. The moths were fed on ripe banana, guava, and pomegranate in small plastic caps inside the cages and the food was replaced daily. Eggs laid were collected and kept in a circular insect-breeding dish until hatching. The eggs were then examined at an interval of 12 h and the emerging larvae were reared individually for the collection of antennae. For antennal transcriptomics, 50 antennae each from unmated male and female moths were excised one day after eclosion using a pair of sterile sharp micro scissors and were immediately frozen in liquid nitrogen, and stored at −80 °C until the RNA was extracted.

### 2.2. RNA Isolation

The RNA was extracted from samples stored using a Direct-zol™ RNA Mini Prep (Cat# R2050, Zymo research, Irvine, CA, USA) kit following the manufacturer’s protocol. In brief, 1 mL of TRI Reagent (Sigma-Aldrich, St. Louis, MO, USA) was added to 100 mg of tissue ground to a fine powder in a DEPC-treated, sterilized mortar and pestle using liquid nitrogen and was vortexed for 30 s. 1 mL of 100% ethanol was then added to the homogenized sample. The sample was loaded into a Zymo-Spin™ IIC column and centrifuged at 12,000× *g*. The initial flow-through was discarded and the column was washed with 500 μL of Direct-zol™ RNA pre-wash buffer. Further, 700 μL of RNA wash buffer was added to the spin tube and centrifuged to discard the flow-through. The RNA was finally eluted in 50 μL of nuclease-free water. The RNA quantity was estimated using Qubit RNA BR assay (Thermo Fisher Scientific, Waltham, MA, USA) and the quality was checked using an Agilent Bio analyzer 2100 (Agilent Technologies, Santa Clara, CA, USA) and RNA Nano kit (Agilent Technologies, Santa Clara, CA, USA).

### 2.3. CDNA Library Construction and RNA Sequencing

Sequencing libraries were prepared from 1 µg of RNA using NEB Next Ultra RNA Library Prep Kit for Illumina (NEB #E7770, New England Biolabs, Ipswich, MA, USA) after depleting the rRNA using an NEB Next rRNA Depletion Kit (Catalog #: E7400S, New England Biolabs, Ipswich, MA, USA). In brief, probes were hybridized to the RNA, treated with RNase H and DNase 1 to degrade the rRNA and DNA probes, and the RNA was purified using Agencourt RNA clean XP. The purified RNA was fragmented and used for cDNA synthesis, which was subsequently PCR enriched, adapter ligated, and purified using 1.8× Agencourt AMPure XP beads. The library quantity was checked using a Qubit dsDNA HS kit (Thermo Fisher Scientific, Waltham, MA, USA). The library quality was checked using an Agilent Bioanalyzer 2100 and Agilent DNA 7500 Kit (Agilent Technologies, Santa Clara, CA, USA).

### 2.4. Illumina Sequencing

After quality inspection, the cDNA libraries were denatured and diluted to Illumina-recommended 1.8 pM concentrations using 0.2 nM NaOH and HT1 reagent, respectively. The denatured and diluted libraries were loaded onto a NexSeq reagent cartridge. Cluster generation and sequencing were performed on an Illumina Next Seq 500 to generate 2 × 150 paired-end reads. Soon after completion of the run, demultiplexed sequence data was collected in FASTQ format and the data quality was checked using FastQC (http://www.bioinformatics.babraham.ac.uk/projects/fastqc/, accessed on 14 June 2017) and MultiQC [36] for base quality distribution, % reads with average Q30 and Q20, and % GC and sequencing adapter contamination. Raw sequence reads were processed to remove adapter sequences and low-quality bases using the Trim-galore (http://www.bioinformatics.babraham.ac.uk/projects/trim_galore/, accessed on 14 June 2017) and Trimmomatic [37] packages. The raw sequence reads are available under the SRA accession PRJNA658216. This Transcriptome Shotgun Assembly project was deposited at DDBJ/EMBL/GenBank under the accession GIUU00000000.

### 2.5. De Novo Transcriptome Assembly and Functional Annotation

Quality-passed reads were assembled using Trinity v2.4.0 [38] with default parameters (Kmer = 25). The assembled contigs were clustered using CD-HIT-EST [39] with 90% sequence similarity. The longest sequence of the clustered contigs was considered as a transcript. The transcripts were annotated by mapping onto NCBI nr, Uniprot–Swissprot, and COG databases using Diamond BLASTp with an e-value cutoff of 1 × 10^−5^. The transcripts that mapped onto the database with an e-value > 1 × 10^−5^ and sequence identity < 70% were rejected. Transcripts with confident annotations were considered as unigenes and were used for downstream analysis. Additionally, KEGG Orthology (KO) annotations were assigned using KAAS (KEGG Automatic Annotation Server) [40].

### 2.6. Gene Ontology and KEGG Pathway Enrichment Analysis for Antennal Transcriptomics

Gene ontology (GO) information for the unigenes was retrieved using the idmapping web application in Uniprot [41]. To check the Biological Process, Cellular Component, and Molecular Functions of the transcripts, we performed GO term enrichment analysis using BINGO [42]. A GO distribution graph was drawn using WEGO [43]. The expressed transcripts were submitted as a multiple FASTA file and the available insect gene sets in KEGG were used as trainer gene sets for annotation using the BBH (bi-directional best hit) method. COG terms annotation was performed by importing the NCBI-NR annotation of each gene into MEGAN.

### 2.7. Data Collection of Olfactory and Chemosensation-Related Sequences of Other Lepidopteran Species

The NCBI protein sequence tool was used to download the sequences of related species. Initially, “Lepidoptera” as a search term was used along with the specific protein type, “odorant-binding proteins”/“odorant receptor”/“pheromone-binding proteins”/“chemosensory proteins”/“antennal-binding proteins”/“sensory neuronal proteins”, at the NCBI database. The list of proteins obtained was further classified based on the other species of the family Noctuidae and closely related families. The entire protein information along with sequences in the FASTA format were downloaded and saved in an Excel sheet. The data were sorted based on the specific protein types, and partial sequences and unrelated sequences were removed. Sequences that had short length were also removed. The insect species were arranged, and the sequences obtained after cleaning were aligned with MUSCLE [44] using the MEGA software [45] and the aligned sequences were saved as MAS files.

### 2.8. Olfactory Gene Identification and Phylogenetic Analyses of Antennal Transcriptome

All candidate OBPs, ORs, CSPs, and SNMPs, and PBPs were manually checked by the BLASTx program at the National Center for Biotechnology Information (NCBI). For contigs with hits against genes of interest, open reading frames (ORFs) were identified and the annotation verified OBP, OR, CSP, SNMP and PBP protein sequences. Orthologs in closely related species of *E. materna* were used to analyze the characteristics of olfactory genes in *E. materna*. Nucleotide sequences of all olfactory genes that were identified from the *E. materna* antennal transcriptomes were named according to sequence homology analysis and numbered arbitrarily. The proteins were aligned using MUSCLE [44] and phylogenetic analysis was done with MEGA using the maximum likelihood method. The test of phylogeny was done using the bootstrap method with 500 replications. The method followed was the Jones–Taylor–Thornton (JTT) model. The tree with the highest log likelihood is shown. The percentage of trees in which the associated taxa clustered together is shown next to the branches is the support value for the cluster. The initial tree(s) for the heuristic search were obtained automatically by applying the Neighbor-Join and BioNJ algorithms to a matrix of pairwise distances estimated using a JTT model, and then selecting the topology with a superior log likelihood value. The tree is drawn to scale, with branch lengths measured in the number of substitutions per site. This analysis involved 200 amino acid sequences and there was a total of 638 positions in the final dataset for the FSMCSP tree, 342 amino acid sequences with a total of 796 positions in the final dataset for the FSMOBP tree, 530 amino acid sequences and a total of 1043 positions in the final dataset for the FSMOR tree, and 27 amino acid sequences and a total of 544 positions in the final dataset for FSMSNMP. For the FSMPB tree, this analysis involved 94 amino acid sequences with a total of 178 positions in the final dataset. Evolutionary analyses were conducted in MEGA X [45]. The Newick tree generated was then transferred to Fig Tree 4 (available from http://tree.bio.ed.ac.uk/software/figtree/, accessed on 29 June 2022) using the bootstrap values for further analysis and figure preparation. The branches were assigned a color based on the clade and the radial phylogeny was obtained. A bootstrap analysis of 500 iterations was performed to evaluate the branch strength of each tree. The support values represented on the branches for a cluster are in percentages based on the bootstrap values. They signify the number of times the cluster was generated in a specified number of iterations. In this case, it would be 500.

### 2.9. Identification of Transposable Element-Related Transcripts

Insects are known to harbor transposable elements, which could integrate and be a part of the genome. We used the transcriptome data generated from the antennal tissue to identify presence of transposable elements in *E. materna*. The annotated transcripts were filtered to identify such elements using text filters “transposon” and “transposable element”. The transcripts identified were sorted based on the read counts.

### 2.10. CDNA Synthesis for Sequence Validation

Antennae were collected from male/female FSM moths as explained earlier, and the RNA was extracted from them using a Qiagen Qiaquick RNA extraction kit. The RNA was quantified and about 1 µg of the total RNA was processed to synthesize cDNA (reverse transcriptase cDNA synthesis kit—Qiagen, Germantown, MD, USA) after DNAse1 treatment. The cDNA was diluted 1:100 and used for PCR. Specific primers were used for each of the genes selected using transcriptome data. The PCR cycling parameters were set at 40 cycles of 95 °C for 1 min, 60 °C for 30 s, and 72 °C for 30 s. The products obtained were confirmed by visualization on 1% agarose gel and a column purified before sequencing.

Eleven genes were chosen from the transcriptome data annotations and these were used for sequence validation by RT-PCR. The transcript sequences were used to design primers, and then PCR was carried out with the cDNA synthesized above. The 11 products obtained were column-purified and Sanger-sequenced using specific primers. The sequences obtained were blasted and we could indeed identify the specific protein hits. The sequences were submitted to NCBI with the accession IDs MW186442–MW186452.

### 2.11. STRING Protein Interacting Network Analysis

To perform the STRING analysis of the FSM olfactory proteins, the translated sequences of the transcripts identified from the antennal transcriptome of FSMs were submitted online using the freely available tool at https://string-db.org/, accessed on 1 June 2021. The scoring parameters are described in [46]. The resulting network was analyzed using the *Bombyx mori* L. genome as the reference. The interactions were obtained and interpreted based on the legends provided in the database. A medium confidence level of 0.4 was used for the settings. The non-interacting nodes were then removed to obtain a clear picture of the interactions.

### 2.12. RNA-Editing Site Prediction

cDNA/transcript sequences for the FSM odorant receptors were obtained from the TRINITY transcriptome sequencing files. They were converted to FASTA format and submitted to two different freely available online prediction tools, namely, AIRLINER [47] (http://α.dmi.unict.it/airliner/, accessed on 1 June 2021) and InosinePredict [48] (http://www.biochem.utah.edu/bass/inosinepredict, accessed on 1 June 2021). AIRLINER is a mathematical modeling-based software program that predicts ADAR-based RNA-editing sites using information from several experimentally determined edited sites. The predictions are probability values assigned to the base with respect to the model. Any value > 0.5 is considered to have a high probability of being edited. The data were further sorted in Excel to obtain the number of sites that had a >0.5 probability. Simple functions such as COUNTIF and sort were used. Similarly, InosinePredict is also a freely available online tool that predicts RNA-editing sites based on the possible outcomes of the four human-based ADARs (hADAR1, hADAR2, hADARd1, and hADARd2) in in vivo data. It provides a percent edited possibility—the higher the percentage, the higher the probability that the site will undergo RNA editing. The results obtained were analyzed, and many sites predicted by both of the tools overlapped.

## 3. Results

### 3.1. Sequencing and Unigene Assembly

A total of 32,660,232 raw reads were obtained from the antenna samples, and the removal of low-quality reads yielded 31,416,578 clean reads with lengths ranging from 100 to 136 bp (Q20 content at 99.31% and Q30 content at 93.48%). The clean reads were assembled into 57,600 transcripts. The minimum and maximum length of the assembled transcripts was 301 nt and 10,724 nt, respectively, with the average length of transcripts at 930.96 nt. The GC content was 44.50% and the number of N50 and N10 transcripts was 1324 and 3311, respectively (Table 1). Among the 68,986 assembled *E. materna* transcripts, a total of 16,476 unigenes could be generated and were within a length range of 200–500 bp (Supplementary Figure S1a). The greatest number of hits to closely related species was to *Amyelois transtiella* W., followed by *B. mori* and *Papilio xuthus* L. (Supplementary Figure S1b).

### 3.2. Functional Annotation

Among the 68,986 assembled *E. materna* transcripts, 19,445 unigenes could be generated and a total of 15,718 unigenes showed hits to unique NCBI non-redundant proteins. Finally, 16,476 unigenes were obtained for 57,600 transcripts from the antennae of *E. materna*. At the “Cellular Components” level, the Cell and Cell Part categories were the most abundant, whereas Symplast, Virion, Synapse, Virion Part, and Cell Junction were the least abundant. The Binding and Catalytic Activity categories accounted for majority of the unigenes at the “Molecular Function” level. The Biological Process category showed maximum assignments to the subcategories of Cellular Process and Metabolic Process (Supplementary Figure S2). Further, the annotation of unigenes using the COG database revealed a total of 8559 unigenes that could be annotated and categorized into 25 functional groups. The two largest groups were Post-Translational Modification, Protein Turnover, Chaperones and Intracellular Trafficking, Secretion, and Vesicular Transport, accounting for 2196 unigenes. The smallest groups were Cell Motility and Nuclear Structure with 10 unigenes each (Supplementary Figure S3).

KAAS server based analysis to explore the unigenes involved in different biological and biochemical pathways revealed 5423 unigenes that could be assigned to various KEGG pathways. Further, KEGG Orthology classifications of the unigenes mapped to five categories, i.e., Cellular Process, Environmental Information Processing, Genetic Information Processing, Human Diseases, and Metabolism and Organismal Systems. Among the KEGG Orthology classifications, Signal Transduction was the most abundant group, whereas Cellular Community-Prokaryotes was the least abundant group (Supplementary Figure S4).

### 3.3. RT-PCR Validation of Transcripts

Sequencing results for products obtained from the cDNA using specific primers designed against transcript sequences generated from the data confirmed that the products were, indeed, transcript sequences obtained from FSM. The blast results were recorded and the identified sequences corroborated with the annotations. We successfully identified OBPs from FSM antennae and were able to successfully validate 11 unigenes by RT-PCR and sequencing (MW186442–MW186452) (Table 1).

### 3.4. Identification of Olfactory Genes

A total of 260 olfactory genes, including 16 odorant-binding proteins (OBPs), some specialized OBPs such as four pheromone-binding proteins (PBPs), 40 antennal-binding proteins (ABPs), including two general odorant-binding proteins (GOBPs), 178 odorant receptors (ORs), 17 chemosensory proteins (CSPs), and five sensory neuron membrane proteins (SNMPs) were identified from the antennal transcriptome of *E. materna*.

### 3.5. Odorant-Binding Proteins

**Figure 1:**
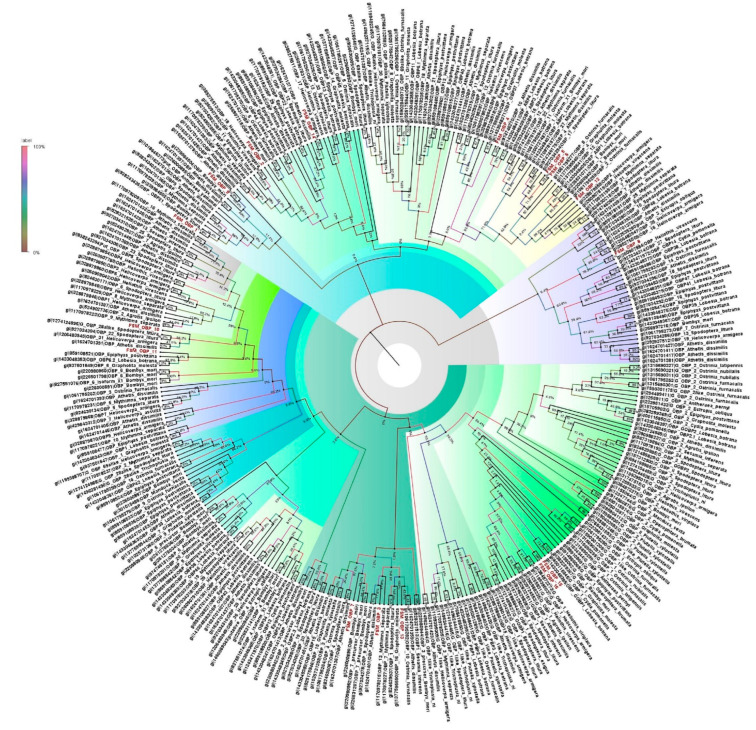
Neighbor-joining dendrogram based on protein sequences of candidate odorant-binding proteins (OBPs) in fruit-sucking moth (FSM) *E. materna*. Phylogenetic analysis of *E. materna* (red) and several related lepidopteran species OBPs was done using the NJ method. The distances generated are based on the bootstrap values. The tree with the highest log likelihood is shown. The percentage of trees in which the associated taxa clustered together is shown next to the branches. FSM OBPs show diversification from each other and other lepidopterans. The colors used were to make the clades more prominent and do not bear any significance to clustering. Confidence values for each tree are represented as percentage values next to the cluster. Values greater than 50% would be considered to have higher confidence levels.

Unigenes corresponding to the annotated olfactory genes (16 OBPs, four PBPs, 40 ABPs) showed similarities to lepidopteran OBPs. All 16 OBPs were almost full length, ranging from 364 to 1540 bp. Besides the entrant OBP genes, some unigenes were identified as GOBP genes in *E. materna* (Supplementary Table S1). The phylogeny obtained from the OBP sequences of several different Lepidoptera species, including members of the Noctuidae family, namely, *E. materna*, *C. punctiferalis*, *Athetis dissimilis* (Hampson), *Helicoverpa armigera* (Hübner), *Heliothes viriplaca* (Hüfnagel), *Spodoptera litura* (Fab.) *Mythimna separata* (Walker) and others, as well as *Epiphyas postvittana* (Walker), *B. mori* and *Manduca sexta* (Linn.) of related families, indicated that most of the time, the identified OBPs of the *E. materna* transcriptome are usually associated with the noctuids. However, there are instances when some of the FSM OBPs (such as FSMOBP5, FSMOBP6, FSMOBP8, and FSMOBP12) were associated with related family moths like *Ostrinia furnacalis* (Guenée). Interestingly, OBPs such as FSMOBP15 and FSMOBP16 were closely associated. Overall, the FSMOBPs are quite diversified and may have developed into specialized proteins responsible for the unusual feeding habits of the moth (Figure 1).

**Figure 2:**
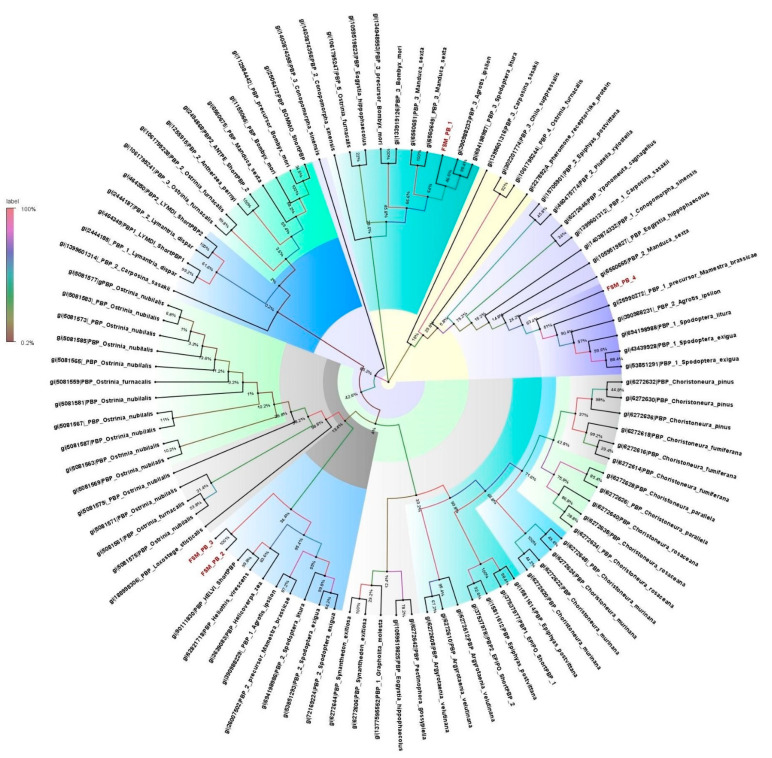
Neighbor-joining dendrogram based on protein sequences of candidate pheromone-binding proteins (PBPs) in fruit-sucking moth (FSM) *E. materna*. Phylogenetic analysis of *E. materna* (red) and several related lepidopteran species PBPs was done using the NJ method. The distances generated are based on the bootstrap values. The tree with the highest log likelihood is shown. The percentage of trees in which the associated taxa clustered together is shown next to the branches. FSM PBPs show diversification from each other and other lepidopterans. The colors used were to make the clades more prominent and do not bear any significance to the clustering. Confidence values for each tree are represented as percentage values next to the cluster. Values greater than 50% would be considered to have higher confidence levels.

The four PBPs identified in the antennal transcriptome of *E. materna* were in the range of 668 to 1517 bp, and a neighbor-joining tree of these sequences with related Lepidoptera species revealed that FSMPB1 and FSMPB4 are associated with PBPs from *M. sexta*, since they occur in the same clade branching off from a distant common ancestor in *C. onopomorpha. sinensis B*. The other PBPs, namely, FSMPB2 and FSMPB3, were closely associated to each other while occurring in the same clade as *Heliothis Virescens* F. and *Loxostege sticticalis* L. (a Crambidae member), respectively (Supplementary Table S2 and Figure 2).

### 3.6. Odorant Receptors

**Figure 3:**
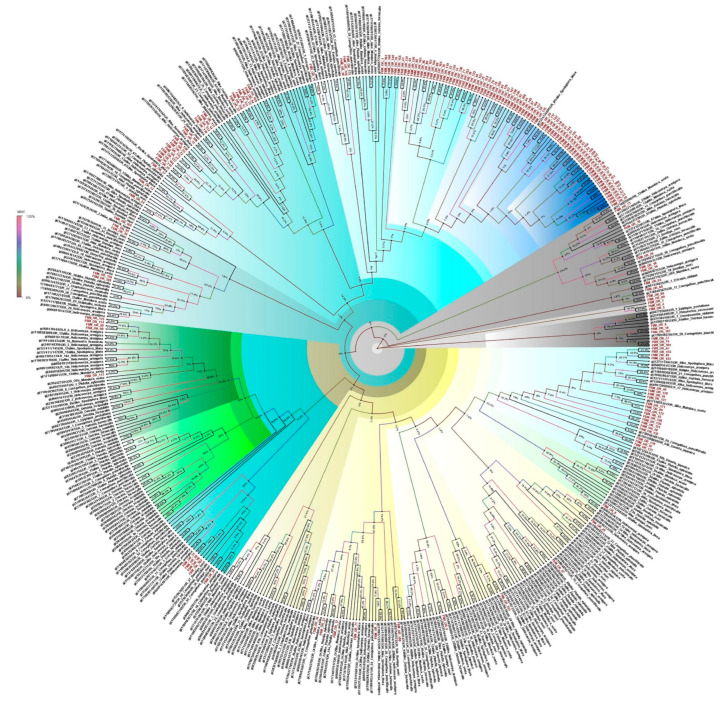
Neighbor-joining dendrogram based on protein sequences of candidate odorant receptors (ORs) in fruit-sucking moth *E. materna*. Phylogenetic analysis of *E. materna* (red) and several related lepidopteran species ORs was done using the NJ method. The distances generated are based on the bootstrap values. The tree with the highest log likelihood is shown. The percentage of trees in which the associated taxa clustered together is shown next to the branches. FSM ORs show diversification from each other and other lepidopterans. There are several ORs that cluster together and are specific to FSM. These could be the ones defining the unusual feeding behavior of FSM. The colors used were to make the clades more prominent and do not bear any significance to the clustering. Confidence values for each tree are represented as percent values next to the cluster. Values greater than 50% would be considered to have higher confidence levels.

Insect ORs are the most important receptors in sex pheromone and general odorant detection processes. A total of 178 ORs were identified from the antennal transcriptome of *E. materna*. The ORs identified are almost complete sequences in the range of 322 bp to 3251 bp. A phylogeny built for the OR sequences revealed that several ORs are closely associated with each other in the sub tree, but only show a relationship with *S. litura*. The tree also provides a display of multiple diversified FSMORs. The closest lepidopteran moth associated with FSM’s ORs is *C. punctiferalis.* With respect to ORs, *E. materna* was found to be closely associated with other moth species such as *C. punctiferalis*, *M. sexta*, *S. litura*, and *O. furnacalis*. The multitude of ORs in FSM might provide the moth with an edge in detecting specific odors, especially at night, and may also help in mate selection (Supplementary Table S3 and Figure 3).

### 3.7. Chemosensory Proteins

**Figure 4:**
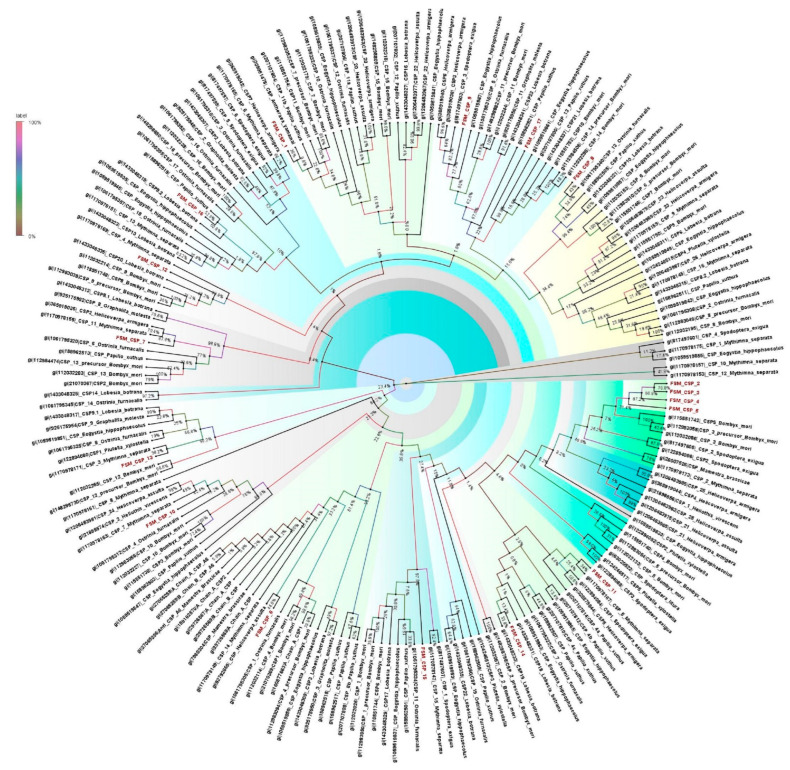
Neighbor-joining dendrogram based on protein sequences of candidate chemosensory proteins (CSPs) in fruit-sucking moth (FSM) *E. materna*. Phylogenetic analysis of *E. materna* (red) and several related lepidopteran species CSPs was done using the NJ method. The distances generated are based on the bootstrap values. The tree with the highest log likelihood is shown. The percentage of trees in which the associated taxa clustered together is shown next to the branches. FSM CSPs show diversification from each other and other lepidopterans. The colors used were to make the clades more prominent and do not bear any significance to clustering. Confidence values for each tree are represented as percent values next to the cluster. Values greater than 50% would be considered to have higher confidence levels.

Insect chemosensory proteins (CSPs) are soluble proteins and widely distributed. CSPs are functionally important for sexual behavior and also the detection of volatiles. In the present study, a total of 17 CSPs were documented from the antennal transcriptome of *E. materna*. The length of the CSPs ranged from 310 bp to 2764 bp (Supplementary Table S4). A phylogeny of the CSP sequences of FSM along with several other Lepidopteran species, such as *B. mori*, *H. virescens*, *H. armigera*, *Helicoverpa assulta* (Guenée), *P. xylostella*, *O. furnacalis*, *Grapholita molesta* (Busck), *Papilio Xuthus* Linn., *S. exigua* (Hübner), *Lobesia botrana* (Denis and Schiffermüller), *Antheraea yamamai* (Guérin-Méneville), *Mamestra brassicae* (Linn.), *S. litura*, *Dendrolimus punctatus* Walk., *M. separata* Walk., *Eogystia hippophaecolus* (Hua, Chou, Fang and Chen), showed divergent relationships with each other. Some specific CSPs such as FSMCSP8 and FSMCSP17 are associated with *O. furnacalis*, *E. hippophaecolus*, *L. botrana* and *B. mori*, but in slightly divergent clades. The other CSPs, such as FSMCSP2, FSMCSP3, FSMCSP 4, and FSMCSP5, clustered together with some divergence (Figure 4). The support values for the tree vary from 100% to very low (4%) in some cases. This is mainly because there are several datapoints and the possibility of some clusters falling together is variable. The confidence would be considered for the clusters that have a support value greater than 50%.

### 3.8. Sensory Neuron Membrane Proteins

**Figure 5:**
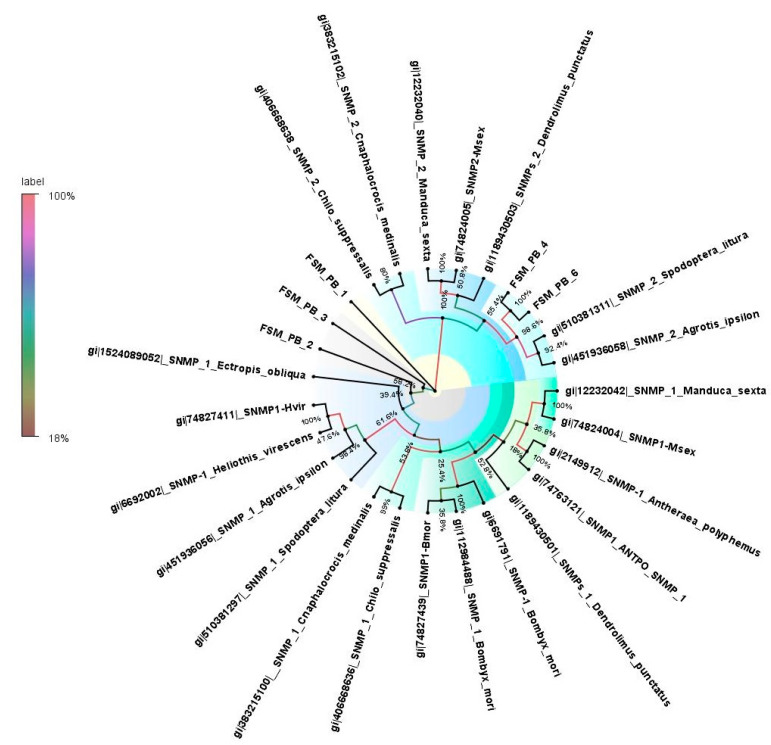
Neighbor-joining dendrogram based on protein sequences of candidate sensory neuron membrane proteins (SNMPs) in fruit-sucking moth (FSM) *E. materna*. Phylogenetic analysis of *E. materna* (red) and several related lepidopteran species SNMPs was done using the NJ method. The distances generated are based on the bootstrap values. The tree with the highest log likelihood is shown. The percentage of trees in which the associated taxa clustered together is shown next to the branches. FSM SNMPs show diversification from each other and other lepidopterans. The colors used were to make the clades more prominent and do not bear any significance to the clustering. Confidence values for each tree are represented as percent values next to the cluster. Values greater than 50% would be considered to have higher confidence levels.

A total of five SNMPs were identified from the antennal transcriptome of *E. materna* with the length in the range of 332 bp to 3217 bp. A neighbor-joining tree of SNMP sequences showed divergent relationships with each other. Of all, FSMSNMP1 is the most likely divergent protein. The SNMPs, namely, FSMSNMP2 and FSMSNMP3, clustered together; however, these SNMPs look significantly divergent based on the branch length. The other SNMPs, namely, FSMSNMP4 and FSMSNMP6, showed associations with *S. litura* and *Agrotis ipsilon* (Hüfnagel) (Supplementary Table S5 and Figure 5). The support values for the tree vary from 100% to very low (4%) in some cases. This is mainly because there are several datapoints and the possibility of some clusters falling together is variable. Moreover, 40 ABPs were identified in the antennal transcriptome of *E. materna*, where ABP27 had the longest assembled sequence (4573 bp) and ABP 31 had the shortest (321 bp) (Supplementary Table S6).

### 3.9. Ionotropic Receptors

**Figure 6:**
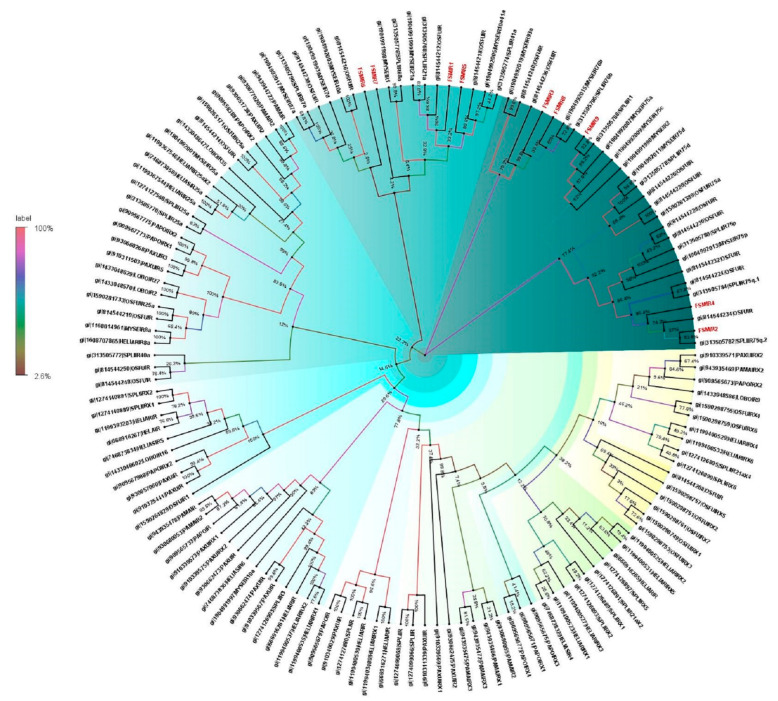
Neighbor-joining dendrogram based on protein sequences of candidate ionotropic receptors (IRs) in fruit-sucking moth (FSM) *E. materna*. Phylogenetic analysis of *E. materna* (red) and several related lepidopteran species IRs was done using the NJ method. The distances generated are based on the bootstrap values. The tree with the highest log likelihood is shown. The percentage of trees in which the associated taxa clustered together is shown next to the branches. FSMIRs show diversification from each other and other lepidopterans. The colors used were to make the clades more prominent and do not bear any significance to clustering. Confidence values for each tree are represented as percent values next to the cluster. Values greater than 50% would be considered to have higher confidence levels.

Ionotropic receptors (IRs) have evolved from ionotropic glutamate receptors, which are a conserved group of synaptic ion ligand-gated ion channels. The antennal transcriptome of *E. materna* helped identify nine IRs. These, when phylogenetically analyzed with IRs from other related insect species, suggested that the FSM IRs clustered with those of *S. litura* (Figure 6) and *O.* *furnacalis*. However, the individual IRs maintained their divergence from each other.

### 3.10. Most-Represented Transcripts

When the identified genes were sorted based on the raw counts, the most-represented members included the ribosomal proteins, followed by cytochrome oxidase subunits; chemosensory protein 3 was among the first 150 genes with high raw counts. Other olfactory related proteins such as CSP5, sensory neuron membrane protein 1, sensory neuron membrane protein 2, take-out protein, and odorant-binding protein 7 were also found among the top 500 genes with high raw counts. The other related genes were general odorant-binding protein 72-like, odorant-binding protein 5, synaptic vesicle glycoprotein 2C-like, sensory neuron membrane protein 1, and synaptic vesicle glycoprotein 2C-like (Supplementary Table S7).

### 3.11. Transposable Elements

Most insects are known to have mobile genetic elements in their genome. Considering the possibility that the antennal tissue might show some interesting features with respect to the occurrence of such elements and their activity, we explored the presence of such elements in the antennal transcriptome of *E materna.* Interestingly, we found hits against several transposable elements reported and identified in lepidopterans. The results are provided in the Supplementary Table S7.

### 3.12. General Transcripts (Non-Olfactory)

Some interesting non-olfactory-related proteins in the first 500 were circadian clock-controlled protein-like, PREDICTED: ecdysteroid-regulated 16 kDa protein-like, no child left behind, bax inhibitor 1 isoform X1, moesin/ezrin/radixin homolog 1 isoform X1, calmodulin, basigin, aquaporin AQPAe.a-like, titin, octopamine receptor in mushroom bodies, hypothetical protein OXYTRI_14248, ribosome-associated membrane protein 4, stomatin-2, adiponectin receptor protein, ankyrin-3-like, glucose dehydrogenase, synaptobrevin-1, facilitated trehalose transported Tret1, and Apolipo proteins (Supplementary Table S7).

### 3.13. RNA-Editing Events in the FSM Odorant Receptors

Compared to the other related species, several odorant receptors were identified in *E. materna*, which is quite interesting. There is evidence in other protein families among insects of RNA-editing events that serves to give rise to protein diversity from a group of genes that have parallel functions, such as those in *B. mori*, mosquitoes, bumblebees, and *Drosophila.* Evidence of any such RNA-editing sites in the multiple ORs identified in the *E. materna* antennal transcriptome would suggest evolutionary significance of multiple ORs identified here. Therefore, freely available online tools such as AIRLINER and InosinePredict were used to predict such sites.

FASTA sequences of 124 FSMORs were submitted to AIRLINER and InosinePredict for the mathematical model-based prediction of RNA-editing sites in each sequence. Interestingly, in the 124 sequences used, the results from AIRLINER provided a total of 8895 sites that had an editing probability of >0.5 (Supplementary Table S8). There were 25 sequences with more than 100 edited sites with >0.5 editing probability. The hADAR1-based prediction in InosinePredict showed 5718 sites that had a prediction of >50% (Supplementary Table S9). Though preliminary, these results suggest that *E. materna* could possibly use the ADAR-dependent RNA-editing process to use ORs for different neurobehavioral, ecological, and chemo-ecological functions. Interestingly, four transcripts identified in the *E. materna* transcriptome had hits to adenosine deaminases that have a known function in RNA-editing events. One of the transcripts, when translated into a protein sequence using the Expasy Translate tool, showed homology to adenosine deaminase protein of several moth species such as *M. sexta*.

**Figure 7:**
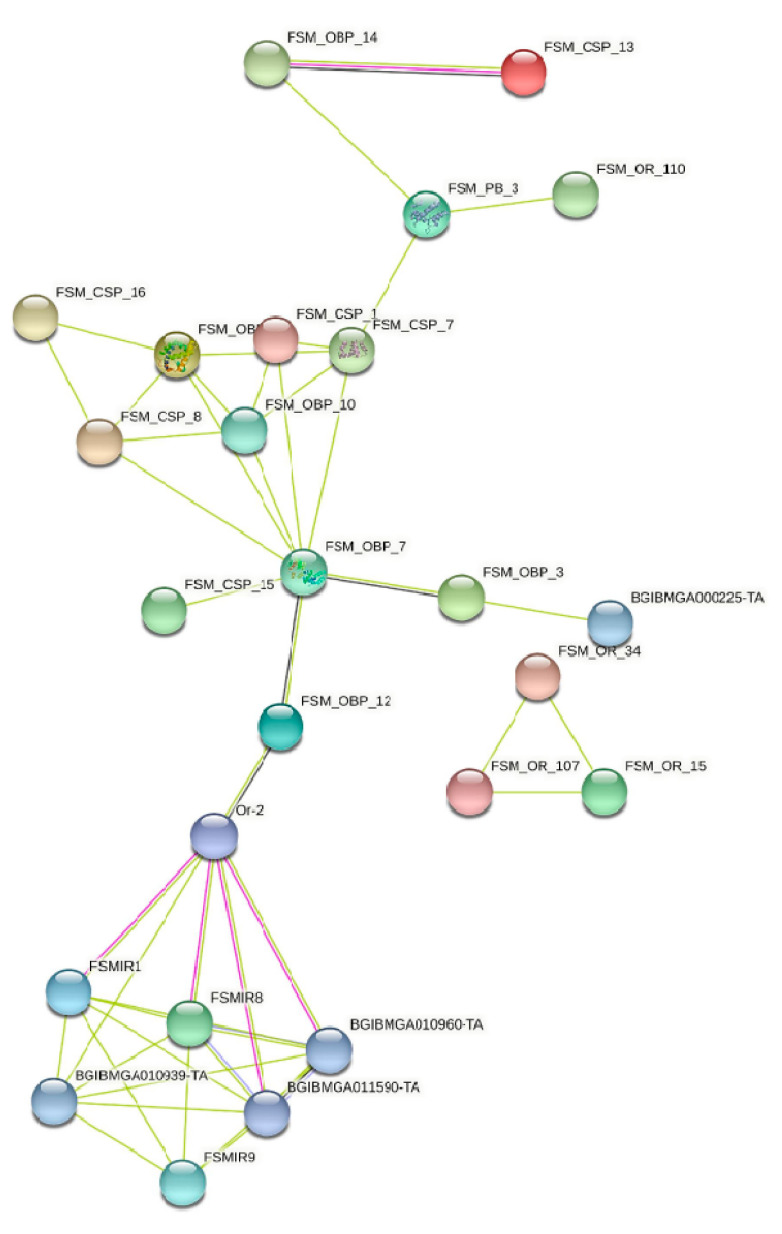

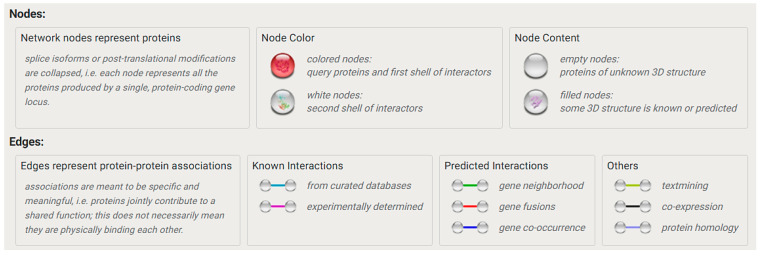
STRING protein interaction network analysis of fruit-sucking moth (FSM) *E. materna* transcripts translated into protein sequences.

STRING protein network analysis of some of the groups of sequences and also among themselves was carried out to explore the possible interactions. CSP proteins from FSM along with OBPs showed very few interactions. The most interesting interaction was between FSMOBP14 (annotated as *B. mori* sercotropin) and FSMCSP13. These proteins show evidence of co-expression and the interaction is experimentally determined based on the edges. The network also shows evidence of co-occurrence for FSM-OBP7 with FSMOBP3 and FSMOBP12. Allowing interacting partners with the *B. mori* proteins at the next level revealed the interaction of FSMIR 8 and FSMIR1 with the *B. mori* Or2, which showed evidence for co-expression with FSMOBP12 (Figure 7).

## 4. Discussion

Insect olfaction is the key area of focus for many agricultural researchers, since insects use olfactory senses to locate host plants, select mates, and detect threats. A molecular understanding of olfaction can help design customized solutions for pest control, particularly in devastating pests such as the fruit-sucking moth *E. materna*. The identification of 19,455 unigenes from 68,986 assembled transcripts and RT-PCR based sequence validation of the samples suggested the corroboration of the good quality of the data generated. The occurrence of a good number of transcripts from the antennal transcriptome suggests a high representation of actively transcribed genes reiterating the active role of the antenna. Antennal transcriptomes of other lepidopterans such as *C. suppressalis* [20] and *C. punctiferalis* [21] yielded a total of 66,560 and 47,109 unigenes, respectively.

The antennal transcriptome data for FSMs were generated with an aim to identify and explore the specific transcripts associated with the antennal tissue that aids the insects’ response to chemo-ecological stimuli. In concurrence with the expectation, the COG categories that were most represented in the transcriptome were Post Translational Modification, Protein Turnover, Chaperones and Intracellular Trafficking, Secretion, and Vesicular Transport. The most-represented KEGG pathways were Environmental Information processing and, specifically, Signal Transduction, suggesting the antennal role in responding to environmental signals. Among the interesting genes identified, the most--represented transcript, Basigin, is known to be important for sensitivity to odors in Drosophila [49]. The interaction of the protein encoded by this gene with Apolipo protein D, a carrier for lipophilic molecules, has been documented [50]. Moreover, aquaporins, which have a suggested role in olfaction [51], were identified in the *E. materna* antennal transcriptome.

In addition to the olfactory-related transcripts and pathways, the identification of other interesting features specific to the FSM would help understand why some of the olfactory genes were found in unusual numbers. Therefore, the presence of transposon-related transcripts was explored in the dataset generated. The occurrence of transposable elements has been reported in several insects [52] and, not surprisingly, transcripts homologous to such elements were also identified in *E. materna*. PiggyBac and pogo-like elements were identified in the *E. materna* antennal transcriptome, which have also been reported in moths such as *P. xylostella* [53] and also some butterflies, including *Papilio* [54]. Surprisingly, transposable element-related results have not been reported in the transcriptomes published earlier for other fruit-sucking moths. The noteworthy aspect of this finding is that they are expressed in the antennal tissue. The occurrence of these transposable elements could reflect the possibility of the horizontal transmission of genetic material occurring in these moths [55]. The horizontal transmission of genetic material by transposable elements has been linked to facilitate genome evolution in insects [56]. Whether the occurrence of these transposable elements in the genome could have facilitated the evolution of olfactory genes is a question that requires insightful studies and deeper understanding.

Data generated in this study were specifically categorized to explore the olfactory-related genes, and different classes were generated including ORs, OBPs, CSPs, SNPs, and IRs. Transcriptome profiles generated for other lepidopteran insects seem to have a lot in common with *E. materna*. The antennal transcriptome of another fruit-sucking moth, *O. emarginata*, was released recently and identified 35 ORs, 41 OBPs, and 20 CSPs [28], against 178 ORs, 16 OBPs, and 17 CSPs in *E. materna*. Surprisingly, fewer ORs and a higher number of OBPs were identified in *O. emarginata*, another Noctuidae fruit-sucking moth, compared to *E. materna*. The number of *E. materna* ORs recognized in this study (178) was significantly more than those identified in other lepidopterans, viz., 46 and59 in *C. punctiferalis* [21,57], 33 in *Dendrolimus kikuchii* G. [58], 47 in *H. armigera* [59], 39 in *Sesamia inferens* W. [60], and 72 in *B. mori* [61]. The OBPs, SNPs, and CSPs identified here were comparable to those identified in closely related moths [20,21,24,57,58,59,62,63,64,65,66]. To compare how the olfactory-related genes of *E. materna* fared with respect to other closely related insects/moths, phylogenetic analysis was carried out for every class using data available from public resources. The phylogenetic trees generated suggested the association of the *E. materna* protein sequences with closely related moth species of the orders Noctuids and Cambridae. The ORs showed a stark difference in numbers, and those in *E. materna* were found to be associated with *Spodoptera litturis*’ OR sequences. The phylogenetic analysis does provide some information about the closeness of *E. materna* to certain insect species, although nothing conclusive can be established in this regard since more detailed sequence information will be needed for the closely associated datasets. The trees generated for each of the olfactory genes show some unique clusters that have good support values and provide hints on the relatedness of the FSM to other moth species, and also provide insights on how different the moth is compared to other closely related moths.

However, the identification of several specialized ORs in the *E. materna* transcriptome could have a bearing on the behavioral patterns such as its unique feeding and egg laying behaviors. To assess this further, an explorative analysis of the OR sequences was carried out to look for any signatures of RNA-editing events that have been reported in the chemosensory proteins of *B. mori* [67]. Studies of Drosophila and other insects involving nAChRs suggest the involvement of RNA editing and highlight its evolutionary significance [68,69]. An RNA-editing mechanism involving the A-I modification has been shown to be important for the proper functioning of the nervous system and its integrity in Drosophila [32]. More recent studies with honey bees suggests the role of A-I RNA editing in adaptation and convergent evolution [70]. The results presented here for the A-I editing sites in FSMORs suggest a similar possibility with regard to odorant reception in *E. materna*. The RNA-editing predictions obtained for FSMORs using the AIRLINER and InosinePredict tools suggest a lot of modifications that occur in these sequences that could possibly result in functional gains. The confidence levels for about 600 sites among the 125 ORs tested was above 75%, and many of the sites overlapped in the results from both prediction tools. As a consequence, the resultant translated proteins could, therefore, have functional diversity and may help the insect specialize. Therefore, the occurrence of several ORs in *E. materna* could possibly be a result of RNA editing.

## 5. Conclusions

The current study is the first to report the antennal transcriptome profile of the fruit-sucking moth *E. materna* and we successfully identified 16 OBPs, four PBPs, 17 CSPs, 178 ORs, and other related transcripts including five SNMPs. Phylogenies constructed for the different categories of genes showed that *E. materna* has evolved olfactory-related genes in different clusters and showed divergence, even to closely related species, possibly reflecting its unique feeding and egg-laying habits. Despite the confidence/support values for some of the clusters being low, there were other prominent clusters that suggest that the moth has evolved to accommodate for its unique feeding habits and life cycle. The RNA-editing events identified also provide support in this regard. The transcriptome data provide information about the actively expressed genes in the antennae, which could be used for knockdown studies for further characterization and also to design customized control measures for other related fruit-sucking moths such as *E. phalonia*, *E. salaminia*, and *E. homaena.* The data presented in this study show evidence, for the first time, for RNA editing in FSMORs. The RNA-editing site data highlight the fact that the occurrence of multiple ORs in FSM could be a result of post-transcriptional RNA-editing events and thus might lead to protein diversity. Such an occurrence could be significant in insect evolution and pest adaptations. The data also provide evidence for the expression of mobile element transcripts in the antennal tissue. A detailed analysis of FSM genes and other related moths will provide further insights into this aspect. The transcriptome of *E. materna* has helped establish a unique and useful resource of information that can support molecular efforts such as RNAi to design long-term solutions to control it. It would be interesting to look at the genes responsible for the oviposition and feeding behavior in female moths to understand the choice of host differentiation. Though the STRING-based network only provides a glimpse of the possibilities, the identification of these interactions, nevertheless, provides important information.

## Figures and Tables

**Table 1:** Validated unigene sequence details.

SNO.	Accession ID	Description	Primer Name	Primer Sequence
1	MW186442	F-BAR domain-only protein 2-like	FSM1F	AACCTACGCATCCCGTACAC
			FSM1R	GCGCAGTACTTCAATGTGGA
2	MW186443	Predicted esterase B-1-like	FSM2F	AAGCAACACCAAGGCCTCTA
			FSM2R	GATACCACTTTGCGCCATTT
3	MW186444	Carboxylic ester hydrolase	FSM3F	CACCTGTTGGTGGTCACAAG
			FSM3R	CGTGGCTCACGACTGACTTA
4	MW186445	Esterase B1	FSM4F	TAGCGTGTTTGATGCCTCTG
			FSM4R	GAAGCAGTTCCGTTTCTTGC
5	MW186446	Acetate esterase-6	FSM5F	GCCCAGTGAAGAGTCAGGAG
			FSM5R	CTTTAAAAGCGCTGGATTCG
6	MW186447	Chemosensory ionotropic receptor75q2	FSM6F	TGCGTGTCAACAAGGAAGTC
			FSM6R	CTCCGGTCTCCATGTGAAAT
7	MW186448	Odorant receptor 4-like	FSM8F	CGACGCAGTTCAATTTGCTA
			FSM8R	AGCGTCACCTATTCCCACAC
8	MW186449	Odorant-binding protein 4	FSM11F	ATTGGGACGATTTGAAGCAG
			FSM11R	GGCATTGATTTTCGTCCAGT
9	MW186450	Uncharacterized protein	FSM12F	CTAGAGCTACGCCCGTGAAC
			FSM12R	TCCCTGCTGCGAAAGTTATT
10	MW186451	Odorant-binding protein	FSM13F	TTTCCTGGGAACAGGTTGTC
			FSM13R	TGTTTCGGACGTCGTTGTAA
11	MW186452	Odorant-binding protein 1	FSM14F	GGCCGTGATCAAATACAGGT
			FSM14R	CTTCAGCCTCGGATTGTAGC

## Data Availability

The data presented in this study has been deposited at the NCBI under the SRA accession PRJNA658216. This Transcriptome Shotgun Assembly project was deposited at DDBJ/EMBL/GenBank under the accession GIUU00000000.

## Supplementary Materials

The following supporting information can be downloaded at: https://www.mdpi.com/article/10.3390/genes13071207/s1, Figure S1a: The size distribution of the assembled unigenes from *Eudocima materna* L. male and female antennal transcriptome; Figure S1b: Top species distribution of unigenes in *E. materna* transcriptome; Figure S2: Gene ontology (GO) classification for the unigenes in male and female *E. materna* antennal transcriptome; Figure S3: Cluster of Orthologous groups (COG) classification. In total, 8559 unigenes were grouped into 25 COG classification Figure S4: KEGG orthology classification of unigenes, were mapped into five categories i.e., Cellular Process, Environmental Information Processing, Genetic Information Processing, Human diseases, Metabolism and Organismal systems; Table S1: Unigenes of candidate odorant binding proteins in *Eudocima materna* L. antennae transcriptome; Table S2: Unigenes of candidate pheromone binding proteins in *Eudocima materna* L. antennae; Table S3: Unigenes of candidate odorant receptors in *Eudocima materna* L. antennae transcriptome; Table S4: Unigenes of candidate chemo sensory proteins in *Eudocima materna* L. antennae transcriptome; Table S5: Unigenes of candidate sensory neuron membrane proteins in *Eudocima materna* L. antennae transcriptome; Table S6: Unigenes of candidate Antennal binding proteins in *Eudocima materna* L. antennae transcriptome; Table S7: Transcriptome annotations and raw counts; Table S8: Results of Airliner; Table S9: Results of Inosine Predict.
