# Supplementary material for: Antennal Transcriptome of the Fruit-Sucking Moth Eudocima materna: Identification of Olfactory Genes and Preliminary Evidence for RNA-Editing Events in Odorant Receptors
Source: Genes (Basel). 2022 Jul 6;13(7):1207. doi: 10.3390/genes13071207 (PMC9323814; doi:10.3390/genes13071207)

Suplementary Figure S1a. The size distribution of the assembled unigenes from *Eudocima materna* L. male and female antennal transcriptome

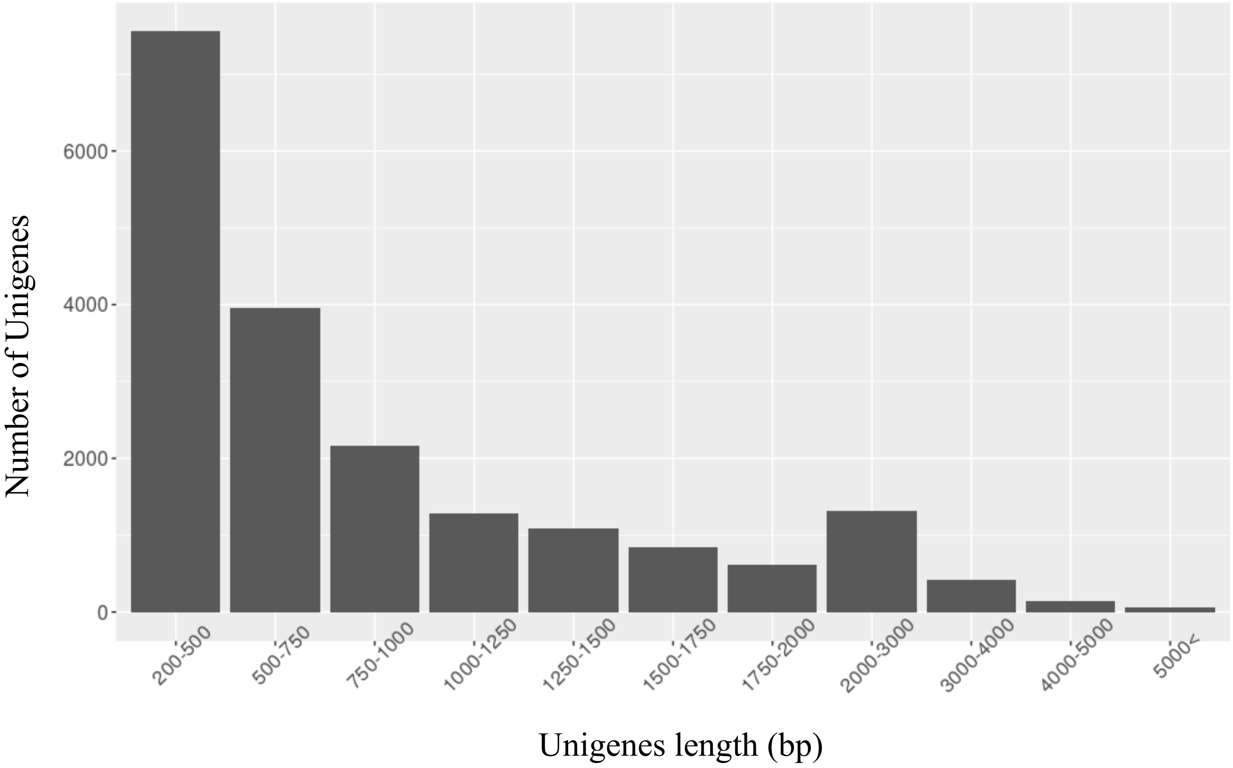

Supplementary Figure S1b. Top species distribution of unigenes in *E. materna* transcriptome

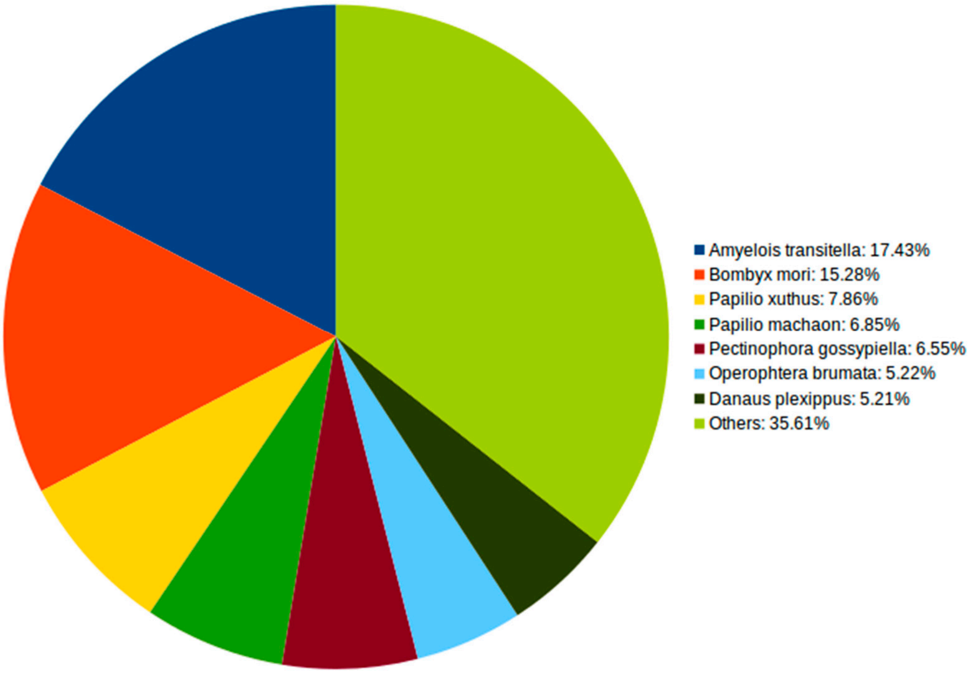
Amyelois transitella: 17.43%
Bombyx mori: 15.28%
Papilio xuthus: 7.86%
Papilio machaon: 6.85%
Pectinophora gossypiella: 6.55%
Operophtera brumata: 5.22%
Danaus plexippus: 5.21%
Others: 35.61%

Supplementary Figure S2. Gene ontology (GO) classification for the unigenes in male and female *E.materna* antennal transcriptome

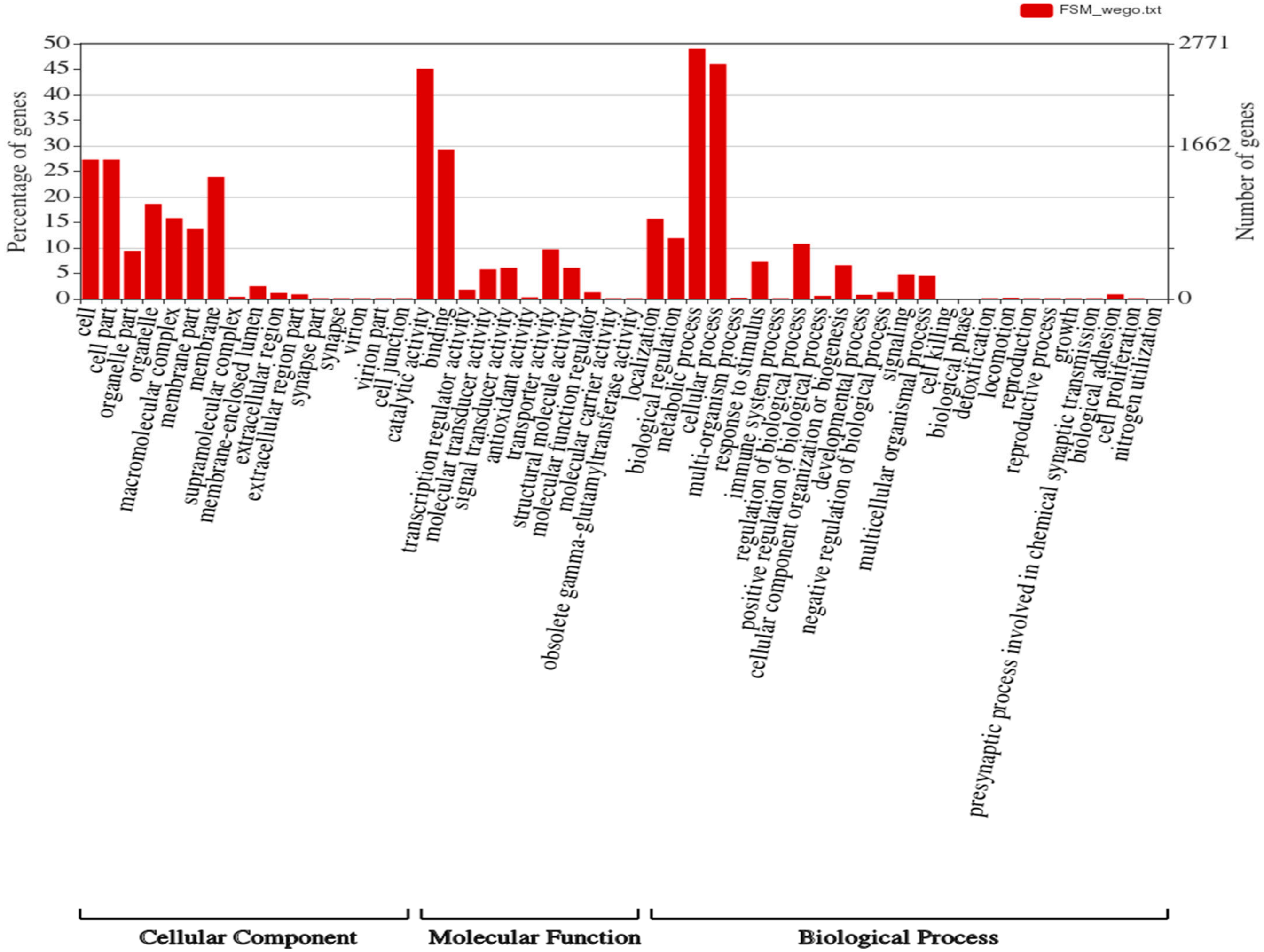

Supplementary Figure S3. Cluster of Orthologous groups (COG) classification. In total, 8,559 unigenes were grouped into 25 COG classifications.

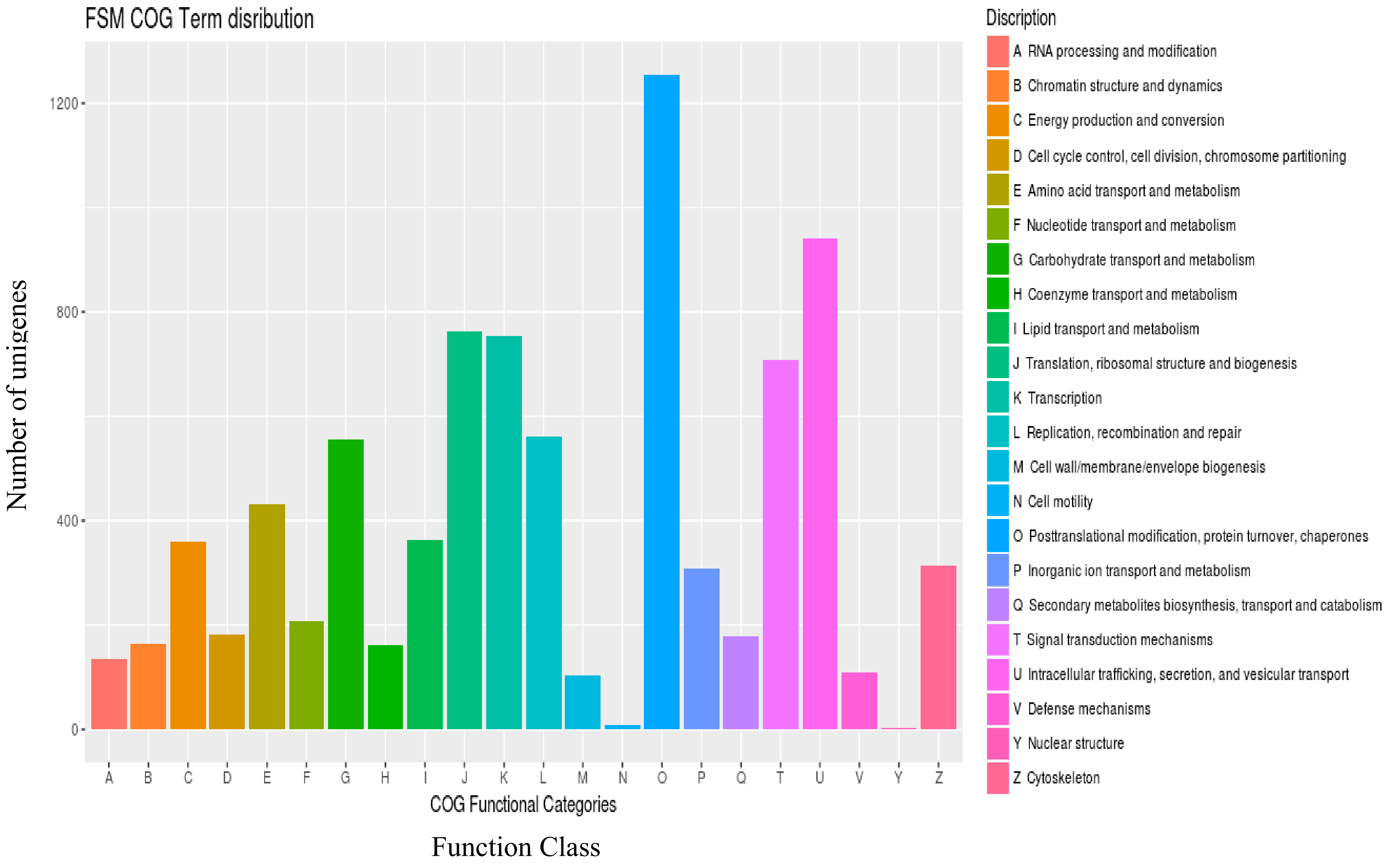

Supplementary Figure S4. KEGG orthology classification of unigenes, were mapped into five categories *i.e.*, Cellular Process, Environmental Information Processing, Genetic Information Processing, Human diseases, Metabolism and Organismal systems

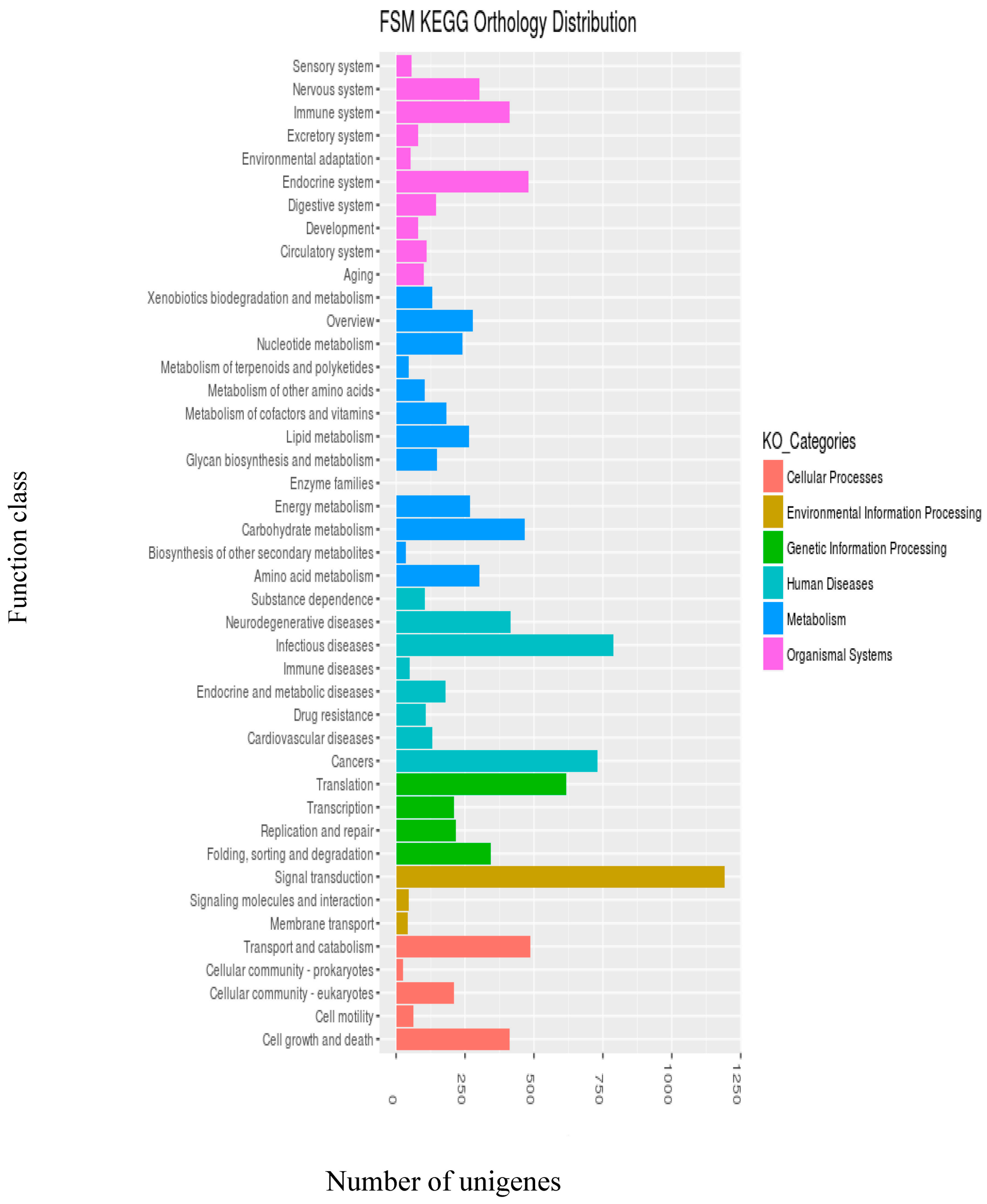

Supplement: Supplementary file 1 [file genes-13-01207-s001.zip › Supplementary Material June 22, 2022/Fruit sucking moth Supplementary Figures 1-4, April 28, 2022.pdf]
