# Supplementary material for: Antennal Transcriptome of the Fruit-Sucking Moth Eudocima materna: Identification of Olfactory Genes and Preliminary Evidence for RNA-Editing Events in Odorant Receptors
Source: Genes (Basel). 2022 Jul 6;13(7):1207. doi: 10.3390/genes13071207 (PMC9323814; doi:10.3390/genes13071207)
Supplement: Supplementary file 1 [file genes-13-01207-s001.zip › Supplementary Material June 22, 2022/Fruit sucking moth Supplementary tables 1-6 April 28, 2022.pdf]

Supplementary Table S1. Unigenes of candidate odorant binding proteins in *Eudocima materna* L. antennae transcriptome.

S. No	Transcript/ Unigene Id.	Gene name	Unigene length (bp)	BLASTx annotation	Score	*E*-value	Identity percent	Count
1	TRINITY_DN22094_c0_g1_i1	FSMOBP1	829	AOG12855.1 Odorant binding protein [*Eogystia hippophaecolus*]	220.7	4.50E-54	50.2	149
2	TRINITY_DN23355_c0_g1_i1	FSMOBP3	1142	BAV56793.1 Odorant binding protein 6 [*Ostrinia furnacalis*]	177.6	6.00E-41	65.3	1189
3	TRINITY_DN23474_c0_g1_i2	FSMOBP4	1101	JAV45900.1 Odorant binding protein 13 [*Mythimna separata*]	384.4	3.10E-103	62.8	914
4	TRINITY_DN24458_c2_g5_i1	FSMOBP5	847	AGP03456.1 Sexi Odorant binding protein 10 [*Spodoptera exigua*]	192.6	1.30E-45	70.2	9020
5	TRINITY_DN24458_c2_g5_i1	FSMOBP6	847	AGP03456.1 Sexi Odorant binding protein 10 [*Spodoptera exigua*]	192.6	1.30E-45	70.2	9020
6	TRINITY_DN24458_c2_g5_i6	FSMOBP7	581	ALZ45420.1 Odorant binding protein 4 [*Athetis dissimilis*]	234.2	2.70E-58	75	25665
7	TRINITY_DN24724_c0_g10_i1	FSMOBP8	742	AKI87965.1 Odorant binding protein 4 [*Spodoptera litura*]	178.7	1.80E-41	53.8	29956
8	TRINITY_DN24724_c0_g2_i1	FSMOBP9	1051	EHJ67714.1 Odorant binding protein [*Danaus plexippus*]	194.9	3.30E-46	60.4	137

9	TRINITY_DN24989_c4_g1_i2	FSMOBP10	1540	AKT26500.1 Odorant binding protein 23 [*Spodoptera exigua*]	379	1.80E-101	66.7	5756
10	TRINITY_DN25157_c2_g1_i2	FSMOBP11	608	AKT26503.1 Odorant binding protein 26 [*Spodoptera exigua*]	217.6	2.80E-53	74.3	8294
11	TRINITY_DN25274_c5_g1_i10	FSMOBP12	1484	AKT26503.1 Odorant binding protein 26 [*Spodoptera exigua*]	70.9	1.00E-08	45.6	767
12	TRINITY_DN25731_c0_g1_i1	FSMOBP13	700	AEB54586.1 Odorant binding protein 2 [*Helicoverpa armigera*]	225.3	1.50E-55	71.1	10505
13	TRINITY_DN25769_c7_g1_i2	FSMOBP14	1239	JAV45906.1 Odorant binding protein 7 [*Mythimna separata*]	275	3.00E-70	63.6	5240
14	TRINITY_DN25987_c3_g1_i1	FSMOBP15	837	AGH70105.1 Odorant binding protein 9 [*Spodoptera exigua*]	250.8	4.10E-63	92.5	21109
15	TRINITY_DN26101_c18_g2_i1	FSMOBP18	1049	AFI25167.1 General odorant binding protein 1 [*Heliothis viriplaca*]	184.5	4.50E-43	80.4	325963
16	TRINITY_DN26101_c18_g2_i2	FSMOBP19	395	AFI25167.1 General odorant binding protein 1 [*Heliothis viriplaca*]	183	4.90E-43	79.4	20307

Note- Above some unigenes are coded by same protein id. but with different reading frames

Supplementary Table S 2. Unigenes of candidate pheromone binding proteins in *Eudocima materna* L. antennae

S. No	Transcript/ Unigene Id.	Gene name	Unigene length (bp)	BLASTx annotation	Score	*E*-value	Identity percent	Count
**1**	TRINITY_DN24431_c5_g1_i2	FSMPBP1	668	ACY78413.1 Pheromone binding protein 3 [*Spodoptera exigua*]	229.2	1.00E-56	62.2	62520
**2**	TRINITY_DN25793_c1_g4_i1	FSMPBP2	1517	AAS55551.2 Pheromone binding protein 2 [*Spodoptera exigua*]	141	8.30E-30	55.7	42521
**3**	TRINITY_DN25793_c1_g4_i2	FSMPBP3	1019	AAC36315.1 Pheromone binding protein [*Helicoverpa zea*]	235	2.80E-58	63.4	66353
**4**	TRINITY_DN25794_c11_g2_i3	FSMPBP4	809	AAC05702.2 Pheromone binding protein 1 precursor [*Mamestra brassicae*]	239.6	9.10E-60	67.7	43875

Supplementary Table S3. Unigenes of candidate odorant receptors in *Eudocima materna* L. antennae transcriptome.

S. No	Transcript/ Unigene Id.	Gene name	Unigene length (bp)	BLASTx annotation	Score	*E*-value	Identity percent	Count
1	TRINITY_DN13291_c0_g1_i1	FSMOR1	694	ABQ84982.1 Putative chemosensory receptor 12 [*Spodoptera littoralis*]	272.3	1.10E-69	52.8	28
2	TRINITY_DN14385_c0_g1_i1	FSMOR2	693	AIG51901.1 Odorant receptor, partial [*Helicoverpa armigera*]	281.6	1.80E-72	73.6	43
3	TRINITY_DN1439_c0_g1_i1	FSMOR3	328	AIG51875.1 Odorant receptor [*Helicoverpa armigera*]	155.2	9.20E-35	76.1	12
4	TRINITY_DN17232_c0_g1_i1	FSMOR4	562	ALM26226.1 Odorant receptor 36, partial [*Athetis dissimilis*]	234.2	2.70E-58	61.9	18
5	TRINITY_DN17232_c0_g1_i2	FSMOR5	422	ALM26226.1 Odorant receptor 36, partial [*Athetis dissimilis*]	141.7	1.30E-30	63.8	8
6	TRINITY_DN18296_c0_g1_i1	FSMOR6	394	XP_012548773.1 Odorant receptor 43a-like [*Bombyx mori*]	132.1	1.00E-27	62.4	10
7	TRINITY_DN18690_c0_g1_i1	FSMOR7	730	AGG08878.1 Putative olfactory receptor 12 [*Spodoptera litura*]	275.8	1.00E-70	65.7	70
8	TRINITY_DN18839_c0_g1_i1	FSMOR8	1335	AIG51875.1 Odorant receptor [*Helicoverpa armigera*]	249.6	1.50E-62	90.4	106
9	TRINITY_DN19460_c0_g1	FSMOR9	1296	AEF32141.1 Odorant receptor [*Spodoptera	615.5	9.80E-	76	107

	_i1			*exigua*]		173		
10	TRINITY_DN19493_c0_g1_i1	FSMOR10	1296	JAV45826.1 Olfactory receptor 39, partial [*Mythimna separata*]	115.9	2.40E-22	65.1	98
11	TRINITY_DN19788_c0_g1_i1	FSMOR11	637	CAD31950.1 Putative chemosensory receptor 9 [*Heliothis virescens*]	184.9	2.10E-43	58.3	40
12	TRINITY_DN20289_c1_g1_i1	FSMOR12	918	AIG51879.1 Odorant receptor [*Helicoverpa armigera*]	459.1	8.30E-126	81.7	99
13	TRINITY_DN20449_c1_g1_i1	FSMOR13	474	JAV45824.1 Olfactory receptor 41 [*Mythimna separata*]	149.8	5.50E-33	65.2	13
14	TRINITY_DN20638_c0_g1_i3	FSMOR14	427	AGK90016.1 Olfactory receptor 9 [*Helicoverpa assulta*]	190.3	3.30E-45	69.1	19
15	TRINITY_DN20848_c0_g1_i2	FSMOR15	1344	ALM26231.1 Odorant receptor 41 [*Athetis dissimilis*]	486.9	5.40E-134	64	67
16	TRINITY_DN21202_c0_g1_i1	FSMOR16	717	AIG51880.1 Odorant receptor, partial [*Helicoverpa armigera*]	141.7	2.30E-30	61.4	863
17	TRINITY_DN21412_c0_g1_i2	FSMOR17	1367	ALM26206.1 Odorant receptor 17 [*Athetis dissimilis*]	478.4	2.00E-131	68.5	95
18	TRINITY_DN21468_c0_g1_i1	FSMOR18	1328	AOE48028.1 Putative odorant receptor OR23 [*Athetis lepigone*]	305.4	2.20E-79	56.5	111
19	TRINITY_DN21468_c0_g1_i2	FSMOR19	1425	AOE48028.1 Putative odorant receptor OR23 [*Athetis lepigone*]	486.1	9.80E-134	61.7	152
20	TRINITY_DN21476_c0_g1	FSMOR20	1536	AII01083.1 Odorant receptor	494.2	3.90E-	57.9	134

	_i1			[*Dendrolimus kikuchii*]		136		
21	TRINITY_DN21963_c0_g1_i3	FSMOR21	1465	AIG51886.1 Odorant receptor [*Helicoverpa armigera*]	485.3	1.70E-133	68.4	200
22	TRINITY_DN22281_c0_g1_i2	FSMOR22	1154	ALM26214.1 Odorant receptor 25 [*Athetis dissimilis*]	263.1	1.10E-66	50.9	124
23	TRINITY_DN22301_c0_g1_i2	FSMOR23	1227	BAH66322.1 Olfactory receptor [*Bombyx mori*]	251.1	4.60E-63	53.7	120
24	TRINITY_DN22595_c1_g2_i1	FSMOR24	1038	ALM26250.1 Odorant receptor 85 [Athetis dissimilis]	325.1	2.10E-85	62.1	80
25	TRINITY_DN23096_c0_g1_i1	FSMOR25	1361	AOE48058.1 Putative odorant receptor OR53 [*Athetis lepigone*]	537.3	3.60E-149	72.6	168
26	TRINITY_DN23549_c0_g2_i1	FSMOR26	1370	JAV45835.1 Olfactory receptor 30, partial [*Mythimna separata*]	394	4.90E-106	52.7	248
27	TRINITY_DN23829_c15_g1_i1	FSMOR27	829	BAH66322.1 Olfactory receptor [*Bombyx mori*]	218.4	2.20E-53	51.9	86
28	TRINITY_DN24093_c8_g2_i1	FSMOR28	1408	AGG08878.1 Putative olfactory receptor 12 [*Spodoptera litura*]	385.2	2.30E-103	52.5	166
29	TRINITY_DN24093_c8_g3_i1	FSMOR29	491	ABQ84982.1 Putative chemosensory receptor 12 [*Spodoptera littoralis*]	157.1	3.60E-35	59.1	39
30	TRINITY_DN24245_c8_g1_i1	FSMOR30	992	AIG51898.1 Odorant receptor [*Helicoverpa armigera*]	279.3	1.30E-71	50.5	365
31	TRINITY_DN24245_c9_g2_i1	FSMOR31	854	AIG51898.1 Odorant receptor [*Helicoverpa armigera*]	196.4	9.30E-47	50.8	58
32	TRINITY_DN24293_c4_g4	FSMOR32	978	CAG38113.1	171.8	2.80E-39	54.2	76

	_i3			Putative chemosensory receptor 12 [*Heliothis virescens*]				
33	TRINITY_DN24472_c1_g1_i1	FSMOR33	724	AJD81575.1 Olfactory receptor 41, partial [*Helicoverpa assulta*]	184.1	4.10E-43	82.9	30
34	TRINITY_DN24472_c1_g1_i2	FSMOR34	1307	AIG51887.1 Odorant receptor [*Helicoverpa armigera*]	614	2.90E-172	76.1	83
35	TRINITY_DN24502_c4_g2_i1	FSMOR35	1295	ABQ84982.1 Putative chemosensory receptor 12 [*Spodoptera littoralis*]	389.8	8.70E-105	51.2	130
36	TRINITY_DN24502_c5_g2_i5	FSMOR36	2140	AGG08878.1 Putative olfactory receptor 12 [*Spodoptera litura*]	248.1	6.80E-62	44.3	344
37	TRINITY_DN24502_c5_g2_i5	FSMOR37	2140	AGG08878.1 Putative olfactory receptor 12 [*Spodoptera litura*]	248.1	6.80E-62	44.3	344
38	TRINITY_DN24502_c5_g2_i7	FSMOR38	1841	AGG08878.1 Putative olfactory receptor 12 [*Spodoptera litura*]	466.1	1.40E-127	51.7	397
39	TRINITY_DN24502_c5_g4_i1	FSMOR39	501	JAV45831.1 Olfactory receptor 34 [*Mythimna separata*]	196.8	4.20E-47	56	30
40	TRINITY_DN24502_c5_g5_i2	FSMOR40	1600	ABQ84982.1 Putative chemosensory receptor 12 [*Spodoptera littoralis*]	456.1	1.20E-124	50.7	815
41	TRINITY_DN24520_c0_g1_i1	FSMOR41	1388	JAV45831.1 Olfactory receptor 34 [*Mythimna separata*]	457.6	3.70E-125	50.1	107
42	TRINITY_DN24520_c5_g3_i1	FSMOR42	1364	AGG08878.1 Putative olfactory receptor 12 [*Spodoptera litura*]	463.4	6.50E-127	52	334
43	TRINITY_DN24520_c5_g5	FSMOR43	1348	ABQ84982.1 Putative chemosensory	293.9	6.80E-76	52	179

	_i4			receptor 12 [*Spodoptera littoralis*]				
44	TRINITY_DN24531_c6_g2_i3	FSMOR44	1339	AOE48064.1 Putative odorant receptor OR59 [*Athetis lepigone*]	434.9	2.50E-118	58.2	104
45	TRINITY_DN24531_c6_g2_i4	FSMOR45	886	AOE48064.1 Putative odorant receptor OR59 [*Athetis lepigone*]	150.6	6.10E-33	66.7	60
46	TRINITY_DN24531_c6_g2_i5	FSMOR46	819	AOE48064.1 Putative odorant receptor OR59 [*Athetis lepigone*]	193.4	7.60E-46	63.6	44
47	TRINITY_DN24531_c6_g5_i1	FSMOR47	1406	AOE48053.1 Putative odorant receptor OR48, partial [*Athetis lepigone*]	323.9	6.40E-85	76.8	100
48	TRINITY_DN24531_c6_g5_i1	FSMOR48	1406	AOE48053.1 Putative odorant receptor OR48, partial [*Athetis lepigone*]	323.9	6.40E-85	76.8	100
49	TRINITY_DN24531_c6_g5_i3	FSMOR49	1369	AIG51873.1 Odorant receptor [*Helicoverpa armigera*]	567	4.20E-158	76	108
50	TRINITY_DN24549_c0_g3_i1	FSMOR50	1234	AGG08878.1 Putative olfactory receptor 12 [*Spodoptera litura*]	469.5	8.30E-129	62.3	229
51	TRINITY_DN24549_c0_g4_i2	FSMOR51	891	AGG08878.1 Putative olfactory receptor 12 [*Spodoptera litura*]	370.5	3.80E-99	59	56
52	TRINITY_DN24549_c0_g6_i1	FSMOR52	1579	AGG08878.1 Putative olfactory receptor 12 [*Spodoptera litura*]	282.3	2.40E-72	49.4	260
53	TRINITY_DN24549_c0_g6_i1	FSMOR53	1579	AGG08878.1 Putative olfactory receptor 12 [*Spodoptera litura*]	282.3	2.40E-72	49.4	260
54	TRINITY_DN24549_c0_g6_i2	FSMOR54	1706	JAV45831.1 Olfactory receptor 34 [*Mythimna separata*]	523.5	6.70E-145	57.3	549

55	TRINITY_DN24589_c0_g9_i1	FSMOR55	409	ALM26214.1 Odorant receptor 25 [*Athetis dissimilis*]	155.6	8.70E-35	55.1	14
56	TRINITY_DN24713_c6_g2_i1	FSMOR56	690	ALM26202.1 Odorant receptor 13, partial [*Athetis dissimilis*]	110.5	5.40E-21	44.4	22
57	TRINITY_DN24713_c7_g2_i1	FSMOR57	715	CAG38113.1 Putative chemosensory receptor 12 [*Heliothis virescens*]	169.1	1.30E-38	62.3	129
58	TRINITY_DN24771_c5_g1_i1	FSMOR58	1631	JAV45831.1 Olfactory receptor 34 [*Mythimna separata*]	266.9	1.10E-67	59.3	399
59	TRINITY_DN24771_c5_g1_i2	FSMOR59	1379	JAV45831.1 Olfactory receptor 34 [*Mythimna separata*]	266.9	9.10E-68	59.3	293
60	TRINITY_DN24771_c5_g1_i2	FSMOR60	1379	JAV45831.1 Olfactory receptor 34 [*Mythimna separata*]	266.9	9.10E-68	59.3	293
61	TRINITY_DN24773_c2_g1_i1	FSMOR61	1389	JAV45831.1 Olfactory receptor 34 [*Mythimna separata*]	464.9	2.30E-127	52.2	201
62	TRINITY_DN24773_c5_g1_i2	FSMOR62	1326	AGG08878.1 Putative olfactory receptor 12 [*Spodoptera litura*]	437.2	4.90E-119	58.7	89
63	TRINITY_DN25045_c6_g1_i5	FSMOR63	1853	AII01109.1 Odorant receptor [*Dendrolimus kikuchii*]	269.2	2.50E-68	36.4	154
64	TRINITY_DN25045_c6_g2_i2	FSMOR64	1577	AGG08878.1 Putative olfactory receptor 12 [*Spodoptera litura*]	485.3	1.90E-133	54.3	200
65	TRINITY_DN25045_c6_g6_i1	FSMOR65	1425	ABQ84982.1 Putative chemosensory receptor 12 [*Spodoptera littoralis*]	399.8	9.30E-108	63.6	93

66	TRINITY_DN25045_c9_g1_i1	FSMOR66	535	JAV45831.1 Olfactory receptor 34 [*Mythimna separata*]	198.4	1.50E-47	73.6	32
67	TRINITY_DN25066_c1_g2_i1	FSMOR67	420	ALM26204.1 Odorant receptor 15 [*Athetis dissimilis*]	174.5	1.90E-40	66.4	13
68	TRINITY_DN25138_c5_g1_i1	FSMOR68	2261	ALM26243.1 Odorant receptor 60 [*Athetis dissimilis*]	317.8	7.40E-83	72.1	577
69	TRINITY_DN25138_c5_g1_i1	FSMOR69	2261	ALM26243.1 Odorant receptor 60 [*Athetis dissimilis*]	317.8	7.40E-83	72.1	577
70	TRINITY_DN25213_c0_g3_i1	FSMOR70	1309	AIG51890.1 Odorant receptor [*Helicoverpa armigera*]	465.3	1.70E-127	54	264
71	TRINITY_DN25308_c4_g2_i1	FSMOR71	1566	JAV45831.1 Olfactory receptor 34 [*Mythimna separata*]	155.2	4.40E-34	67.6	167
72	TRINITY_DN25371_c1_g2_i2	FSMOR72	1150	AIG51860.1 Odorant receptor [*Helicoverpa armigera*]	325.9	1.40E-85	50.3	88
73	TRINITY_DN25390_c6_g1_i3	FSMOR73	1695	AII01072.1 Odorant receptor [*Dendrolimus houi*]	328.9	2.40E-86	61.3	470
74	TRINITY_DN25390_c6_g2_i1	FSMOR74	578	JAV45834.1 Olfactory receptor 31, partial [*Mythimna separata]*	273.1	5.30E-70	63	45
75	TRINITY_DN25474_c1_g1_i1	FSMOR75	1915	ABQ84982.1 Putative chemosensory receptor 12 [*Spodoptera littoralis*]	446.4	1.20E-121	56.3	173
76	TRINITY_DN25474_c1_g1_i2	FSMOR76	1780	ABQ84982.1 Putative chemosensory receptor 12 [*Spodoptera littoralis*]	354.4	5.60E-94	48.6	152
77	TRINITY_DN25474_c1_g1	FSMOR77	1780	ABQ84982.1 Putative chemosensory	354.4	5.60E-94	48.6	152

	_i2			receptor 12 [*Spodoptera littoralis*]				
78	TRINITY_DN25474_c1_g1_i8	FSMOR78	1783	ABQ84982.1 Putative chemosensory receptor 12 [*Spodoptera littoralis*]	380.2	9.50E-102	62.2	128
79	TRINITY_DN25486_c0_g1_i2	FSMOR79	433	AGG08878.1 Putative olfactory receptor 12 [*Spodoptera litura*]	144.8	1.60E-31	53.5	16
80	TRINITY_DN25486_c1_g1_i1	FSMOR80	1494	JAV45831.1 Olfactory receptor 34 [*Mythimna separata*]	422.2	1.80E-114	48.1	546
81	TRINITY_DN25486_c1_g2_i3	FSMOR81	881	ABQ84982.1 Putative chemosensory receptor 12 [*Spodoptera littoralis*]	297	5.20E-77	58.4	268
82	TRINITY_DN25486_c1_g5_i1	FSMOR82	987	JAV45831.1 Olfactory receptor 34 [*Mythimna separata*]	189.5	1.30E-44	65.5	74
83	TRINITY_DN25486_c1_g8_i1	FSMOR83	498	AJD81541.1 Olfactory receptor 1, partial [*Helicoverpa assulta*]	177.2	3.40E-41	54.5	17
84	TRINITY_DN25486_c1_g9_i1	FSMOR84	401	JAV45831.1 Olfactory receptor 34 [*Mythimna separata*]	182.6	6.50E-43	70.1	33
85	TRINITY_DN25504_c1_g1_i1	FSMOR85	483	ABQ84982.1 Putative chemosensory receptor 12 [*Spodoptera littoralis*]	179.1	8.70E-42	53.1	103
86	TRINITY_DN25504_c1_g2_i4	FSMOR86	1660	ABQ84982.1 Putative chemosensory receptor 12 [*Spodoptera littoralis*]	439.5	1.20E-119	49	1102
87	TRINITY_DN25504_c1_g3_i2	FSMOR87	583	AJD81541.1 Olfactory receptor 1, partial [*Helicoverpa assulta*]	153.3	6.20E-34	76.3	133

88	TRINITY_DN25504_c1_g4_i2	FSMOR88	861	ABQ84982.1 Putative chemosensory receptor 12 [*Spodoptera littoralis*]	235.3	1.80E-58	57.4	45
89	TRINITY_DN25504_c1_g5_i1	FSMOR89	1239	ABQ84982.1 Putative chemosensory receptor 12 [*Spodoptera littoralis*]	379.8	8.60E-102	52.5	155
90	TRINITY_DN25539_c0_g2_i1	FSMOR90	1473	AIG51898.1 Odorant receptor [*Helicoverpa armigera*]	419.5	1.20E-113	52.3	497
91	TRINITY_DN25539_c0_g5_i1	FSMOR91	1425	JAV45856.1 Olfactory receptor 9 [*Mythimna separata*]	506.1	9.20E-140	58.4	702
92	TRINITY_DN25552_c7_g1_i5	FSMOR92	2764	AGG08878.1 Putative olfactory receptor 12 [*Spodoptera litura*]	298.9	4.30E-77	54.8	1400
93	TRINITY_DN25552_c7_g1_i5	FSMOR93	2764	AGG08878.1 Putative olfactory receptor 12 [*Spodoptera litura*]	298.9	4.30E-77	54.8	1400
94	TRINITY_DN25720_c5_g1_i1	FSMOR94	340	CAG38113.1 Putative chemosensory receptor 12 [*Heliothis virescens*]	149.4	5.20E-33	67.3	8
95	TRINITY_DN25930_c0_g5_i1	FSMOR95	511	CAG38118.1 Putative chemosensory receptor 17 [*Heliothis virescens*]	204.9	1.60E-49	64.7	98
96	TRINITY_DN25930_c0_g6_i2	FSMOR96	787	AOE48064.1 Putative odorant receptor OR59 *[Athetis lepigone*]	252.3	1.30E-63	52.9	125
97	TRINITY_DN25963_c5_g1_i2	FSMOR97	1246	AGY14579.2 Putative odorant receptor [*Sesamia inferens*]	416	1.10E-112	59.1	37
98	TRINITY_DN25963_c5_g1_i3	FSMOR98	963	AGY14579.2 Putative odorant receptor [*Sesamia inferens*]	287	5.90E-74	66.2	28

99	TRINITY_DN26013_c2_g1_i1	FSMOR99	3251	AOE48025.1 Putative odorant receptor OR20 [*Athetis lepigone*]	550.8	7.40E-153	63.3	433
100	TRINITY_DN26722_c0_g1_i1	FSMOR100	398	AOE48066.1 Putative odorant receptor OR61 [*Athetis lepigone*]	183.3	3.80E-43	62.9	12
101	TRINITY_DN31002_c0_g1_i1	FSMOR101	650	JAV45840.1 Olfactory receptor 25 [*Mythimna separata*]	274.2	2.70E-70	73	38
102	TRINITY_DN3116_c0_g1_i1	FSMOR102	1180	ALM26205.1 Odorant receptor 16 [*Athetis dissimilis*]	583.6	3.80E-163	77.7	118
103	TRINITY_DN5592_c0_g1_i1	FSMOR103	322	JAV45826.1 Olfactory receptor 39, partial [*Mythimna separata*]	124.8	1.30E-25	63.7	9
104	TRINITY_DN9513_c0_g1_i1	FSMOR104	535	ALM26219.1 Odorant receptor 30 [*Athetis dissimilis*]	203.4	4.80E-49	53.9	38
105	TRINITY_DN13291_c0_g1_i1	FSMOR105	694	ABQ84982.1 Putative chemosensory receptor 12 [*Spodoptera littoralis*]	272.3	1.10E-69	52.8	28
106	TRINITY_DN18690_c0_g1_i1	FSMOR106	730	AGG08878.1 Putative olfactory receptor 12 [*Spodoptera litura*]	275.8	1.00E-70	65.7	70
107	TRINITY_DN19493_c0_g1_i1	FSMOR107	1296	JAV45826.1 Olfactory receptor 39, partial [*Mythimna separata*]	115.9	2.40E-22	65.1	98
108	TRINITY_DN19788_c0_g1_i1	FSMOR108	637	CAD31950.1 Putative chemosensory receptor 9 [*Heliothis virescens*]	184.9	2.10E-43	58.3	40
109	TRINITY_DN20556_c0_g1_i1	FSMOR109	1694	CAG38119.1 Putative chemosensory receptor 18 [*Heliothis virescens*]	496.5	8.70E-137	61.5	1266

110	TRINITY_DN20638_c0_g1_i2	FSMOR110	493	ACC63237.1 Olfactory receptor 9 [*Helicoverpa armigera*]	141.4	2.00E-30	66.1	11
111	TRINITY_DN20638_c0_g1_i3	FSMOR111	427	AGK90016.1 Olfactory receptor 9 [*Helicoverpa assulta*]	190.3	3.30E-45	69.1	19
112	TRINITY_DN20638_c0_g2_i1	FSMOR112	1035	ACC63237.1 Olfactory receptor 9 [*Helicoverpa armigera*]	331.6	2.30E-87	57.3	70
113	TRINITY_DN21304_c0_g1_i1	FSMOR113	828	CAG38116.1 Putative chemosensory receptor 15 [*Heliothis virescens*]	246.5	7.60E-62	50.8	131
114	TRINITY_DN22301_c0_g1_i2	FSMOR114	1227	BAH66322.1 Olfactory receptor [*Bombyx mori*]	251.1	4.60E-63	53.7	120
115	TRINITY_DN23829_c15_g1_i1	FSMOR115	829	BAH66322.1 Olfactory receptor [*Bombyx mori*]	218.4	2.20E-53	51.9	86
116	TRINITY_DN24093_c8_g2_i1	FSMOR116	1408	AGG08878.1 Putative olfactory receptor 12 [*Spodoptera litura*]	385.2	2.30E-103	52.5	166
117	TRINITY_DN24093_c8_g3_i1	FSMOR117	491	ABQ84982.1 Putative chemosensory receptor 12 [*Spodoptera littoralis*]	157.1	3.60E-35	59.1	39
118	TRINITY_DN24293_c4_g4_i3	FSMOR118	978	CAG38113.1 Putative chemosensory receptor 12 [*Heliothis virescens*]	171.8	2.80E-39	54.2	76
119	TRINITY_DN24502_c4_g2_i1	FSMOR119	1295	ABQ84982.1 Putative chemosensory receptor 12 [*Spodoptera littoralis*]	389.8	8.70E-105	51.2	130
120	TRINITY_DN24502_c5_g2	FSMOR120	644	ABQ84982.1 Putative chemosensory	173.7	4.90E-40	50.6	46

	_i3			receptor 12 [*Spodoptera littoralis*]				
121	TRINITY_DN24502_c5_g2_i7	FSMOR121	1841	AGG08878.1 Putative olfactory receptor 12 [*Spodoptera litura*]	466.1	1.40E-127	51.7	397
122	TRINITY_DN24502_c5_g4_i1	FSMOR122	501	JAV45831.1 Olfactory receptor 34 [*Mythimna separata*]	196.8	4.20E-47	56	30
123	TRINITY_DN24502_c5_g5_i2	FSMOR123	1600	ABQ84982.1 Putative chemosensory receptor 12 [*Spodoptera littoralis*]	456.1	1.20E-124	50.7	815
124	TRINITY_DN24520_c0_g1_i1	FSMOR124	1388	JAV45831.1 Olfactory receptor 34 [*Mythimna separata*]	457.6	3.70E-125	50.1	107
125	TRINITY_DN24520_c5_g3_i1	FSMOR125	1364	AGG08878.1 Putative olfactory receptor 12 [*Spodoptera litura*]	463.4	6.50E-127	52	334
126	TRINITY_DN24520_c5_g5_i4	FSMOR126	1348	ABQ84982.1 Putative chemosensory receptor 12 [*Spodoptera littoralis*]	293.9	6.80E-76	52	179
127	TRINITY_DN24531_c6_g2_i3	FSMOR127	1339	AOE48064.1 Putative odorant receptor OR59 [*Athetis lepigone*]	434.9	2.50E-118	58.2	104
128	TRINITY_DN24531_c6_g2_i4	FSMOR128	886	AOE48064.1 Putative odorant receptor OR59 [*Athetis lepigone*]	150.6	6.10E-33	66.7	60
129	TRINITY_DN24531_c6_g2_i5	FSMOR129	819	AOE48064.1 Putative odorant receptor OR59 [*Athetis lepigone*]	193.4	7.60E-46	63.6	44
130	TRINITY_DN24531_c6_g3_i1	FSMOR130	1561	AGK90020.1 Olfactory receptor 17 [*Helicoverpa assulta*]	360.9	5.20E-96	51.4	201
131	TRINITY_DN24531_c6_g3_i3	FSMOR131	1670	AGK90020.1 Olfactory receptor 17 [*Helicoverpa assulta*]	333.6	9.60E-88	52.1	229

132	TRINITY_DN24531_c6_g5_i1	FSMOR132	1406	AOE48053.1 Putative odorant receptor OR48, partial [*Athetis lepigone*]	323.9	6.40E-85	76.8	100
133	TRINITY_DN24531_c6_g5_i1	FSMOR133	1406	AOE48053.1 Putative odorant receptor OR48, partial [*Athetis lepigone*]	323.9	6.40E-85	76.8	100
134	TRINITY_DN24531_c6_g5_i3	FSMOR134	1369	AIG51873.1 Odorant receptor [*Helicoverpa armigera*]	567	4.20E-158	76	108
135	TRINITY_DN24549_c0_g3_i1	FSMOR135	1234	AGG08878.1 Putative olfactory receptor 12 [*Spodoptera litura*]	469.5	8.30E-129	62.3	229
136	TRINITY_DN24549_c0_g4_i2	FSMOR136	891	AGG08878.1 Putative olfactory receptor 12 [*Spodoptera litura*]	370.5	3.80E-99	59	56
137	TRINITY_DN24549_c0_g6_i2	FSMOR137	1706	JAV45831.1 Olfactory receptor 34 [*Mythimna separata*]	523.5	6.70E-145	57.3	549
138	TRINITY_DN24589_c0_g9_i1	FSMOR138	409	ALM26214.1 Odorant receptor 25 [*Athetis dissimilis*]	155.6	8.70E-35	55.1	14
139	TRINITY_DN24678_c6_g3_i1	FSMOR139	2115	ACL81188.1 Putative olfactory receptor 18 [*Mamestra brassicae*]	503.8	6.80E-139	62.2	1107
140	TRINITY_DN24713_c6_g2_i1	FSMOR140	690	ALM26202.1 Odorant receptor 13, partial [*Athetis dissimilis*]	110.5	5.40E-21	44.4	22
141	TRINITY_DN24713_c7_g2_i1	FSMOR141	715	CAG38113.1 Putative chemosensory receptor 12 [*Heliothis virescens*]	169.1	1.30E-38	62.3	129
142	TRINITY_DN24771_c5_g1_i1	FSMOR142	1631	JAV45831.1 Olfactory receptor 34 [*Mythimna separata*]	266.9	1.10E-67	59.3	399
143	TRINITY_DN24771_c5_g1	FSMOR143	1379	JAV45831.1	266.9	9.10E-68	59.3	293

	_i2			Olfactory receptor 34 [*Mythimna separata*]				
144	TRINITY_DN24773_c2_g1_i1	FSMOR144	1389	JAV45831.1 Olfactory receptor 34 [*Mythimna separata*]	464.9	2.30E-127	52.2	201
145	TRINITY_DN24773_c5_g1_i2	FSMOR145	1326	AGG08878.1 Putative olfactory receptor 12 [*Spodoptera litura*]	437.2	4.90E-119	58.7	89
146	TRINITY_DN25045_c6_g1_i3	FSMOR146	2006	AGG08878.1 Putative olfactory receptor 12 [*Spodoptera litura*]	500.7	5.40E-138	55.8	153
147	TRINITY_DN25045_c6_g1_i5	FSMOR147	1853	AII01109.1 Odorant receptor [*Dendrolimus kikuchii*]	269.2	2.50E-68	36.4	154
148	TRINITY_DN25045_c6_g1_i6	FSMOR148	1270	AGG08878.1 Putative olfactory receptor 12 [*Spodoptera litura*]	407.9	3.00E-110	52.5	69
149	TRINITY_DN25045_c6_g2_i2	FSMOR149	1577	AGG08878.1 Putative olfactory receptor 12 [*Spodoptera litura*]	485.3	1.90E-133	54.3	200
150	TRINITY_DN25045_c6_g6_i1	FSMOR150	1425	ABQ84982.1 Putative chemosensory receptor 12 [*Spodoptera littoralis*]	399.8	9.30E-108	63.6	93
151	TRINITY_DN25045_c9_g1_i1	FSMOR151	535	JAV45831.1 Olfactory receptor 34 [*Mythimna separata*]	198.4	1.50E-47	73.6	32
152	TRINITY_DN25308_c4_g2_i1	FSMOR152	1566	JAV45831.1 Olfactory receptor 34 [*Mythimna separata*]	155.2	4.40E-34	67.6	167
153	TRINITY_DN25474_c1_g1_i1	FSMOR153	1915	ABQ84982.1 Putative chemosensory receptor 12 [*Spodoptera littoralis*]	446.4	1.20E-121	56.3	173
154	TRINITY_DN25474_c1_g1_i8	FSMOR154	1783	ABQ84982.1 Putative chemosensory receptor 12 [*Spodoptera	380.2	9.50E-102	62.2	128

				littoralis]				
155	TRINITY_DN25486_c0_g1_i2	FSMOR155	433	AGG08878.1 Putative olfactory receptor 12 [*Spodoptera litura*]	144.8	1.60E-31	53.5	16
156	TRINITY_DN25486_c1_g2_i3	FSMOR156	881	ABQ84982.1 Putative chemosensory receptor 12 [*Spodoptera littoralis*]	297	5.20E-77	58.4	268
157	TRINITY_DN25486_c1_g3_i1	FSMOR157	1222	AGG08878.1 Putative olfactory receptor 12 [*Spodoptera litura*]	453	7.90E-124	53.6	284
158	TRINITY_DN25486_c1_g5_i1	FSMOR158	987	JAV45831.1 Olfactory receptor 34 [*Mythimna separata*]	189.5	1.30E-44	65.5	74
159	TRINITY_DN25486_c1_g9_i1	FSMOR159	401	JAV45831.1 Olfactory receptor 34 [*Mythimna separata*]	182.6	6.50E-43	70.1	33
160	TRINITY_DN25504_c0_g1_i2	FSMOR160	1147	AJD81541.1 Olfactory receptor 1, partial [*Helicoverpa assulta*]	161.4	4.50E-36	54.2	65
161	TRINITY_DN25504_c0_g1_i2	FSMOR161	1147	AJD81541.1 Olfactory receptor 1, partial [*Helicoverpa assulta*]	161.4	4.50E-36	54.2	65
162	TRINITY_DN25504_c0_g1_i3	FSMOR162	630	JAV45831.1 Olfactory receptor 34 [*Mythimna separata*]	226.5	6.20E-56	52.2	13
163	TRINITY_DN25504_c1_g1_i1	FSMOR163	483	ABQ84982.1 Putative chemosensory receptor 12 [*Spodoptera littoralis*]	179.1	8.70E-42	53.1	103
164	TRINITY_DN25504_c1_g3_i2	FSMOR164	583	AJD81541.1 Olfactory receptor 1, partial [*Helicoverpa assulta*]	153.3	6.20E-34	76.3	133
165	TRINITY_DN25504_c1_g4_i1	FSMOR165	920	ABQ84982.1 Putative chemosensory receptor 12 [*Spodoptera littoralis*]	323.2	7.10E-85	51	45

166	TRINITY_DN25504_c1_g4_i2	FSMOR166	861	ABQ84982.1 Putative chemosensory receptor 12 [*Spodoptera littoralis*]	235.3	1.80E-58	57.4	45
167	TRINITY_DN25504_c1_g5_i1	FSMOR167	1239	ABQ84982.1 Putative chemosensory receptor 12 [*Spodoptera littoralis*]	379.8	8.60E-102	52.5	155
168	TRINITY_DN25552_c7_g1_i5	FSMOR168	2764	AGG08878.1 Putative olfactory receptor 12 [*Spodoptera litura*]	298.9	4.30E-77	54.8	1400
169	TRINITY_DN25720_c5_g1_i1	FSMOR169	340	CAG38113.1 Putative chemosensory receptor 12 [*Heliothis virescens*]	149.4	5.20E-33	67.3	8
170	TRINITY_DN25772_c6_g2_i1	FSMOR170	1378	ABQ84982.1 Putative chemosensory receptor 12 [*Spodoptera littoralis*]	436.8	6.60E-119	52.5	208
171	TRINITY_DN25930_c0_g5_i1	FSMOR171	511	CAG38118.1 Putative chemosensory receptor 17 [*Heliothis virescens*]	204.9	1.60E-49	64.7	98
172	TRINITY_DN25930_c0_g5_i2	FSMOR172	355	CAG38118.1 Putative chemosensory receptor 17 [*Heliothis virescens*]	112.5	7.40E-22	57	24
173	TRINITY_DN25930_c0_g6_i2	FSMOR173	787	AOE48064.1 Putative odorant receptor OR59 [*Athetis lepigone*]	252.3	1.30E-63	52.9	125
174	TRINITY_DN25963_c5_g1_i2	FSMOR174	1246	AGY14579.2 Putative odorant receptor [*Sesamia inferens*]	416	1.10E-112	59.1	37
175	TRINITY_DN25963_c5_g1_i3	FSMOR175	963	AGY14579.2 Putative odorant receptor [*Sesamia inferens*]	287	5.90E-74	66.2	28

176	TRINITY_DN27886_c0_g1_i1	FSMOR176	353	AGG08878.1 Putative olfactory receptor 12 [*Spodoptera litura*]	129	7.50E-27	51.3	8
177	TRINITY_DN5592_c0_g1_i1	FSMOR177	322	JAV45826.1 Olfactory receptor 39, partial [*Mythimna separata*]	124.8	1.30E-25	63.7	9
178	TRINITY_DN605_c0_g1_i1	FSMOR178	485	AGK90007.1 Olfactory receptor 17 [*Helicoverpa armigera*]	156.8	4.60E-35	51.3	15

Note- Above some unigenes are coded by same protein id. but with different reading frames

Supplementary Table S4. Unigenes of candidate chemo sensory proteins in *Eudocima materna* L. antennae transcriptome.

S. No	Transcript/ Unigene Id.	Gene name	Unigene length (bp)	BLASTx annotation	Score	*E*-value	Identity percent	Count
1	TRINITY_DN21089_c0_g1_i1	FSMCSP1	499	AKT26481.1 Chemosensory protein 4 [*Spodoptera exigua*]	159.1	9.70E-36	68.4	42
2	TRINITY_DN23308_c0_g1_i1	FSMCSP2	644	ALJ30213.1 Putative chemosensory protein CSP2 [*Spodoptera litura*]	191.8	1.70E-45	80	510
3	TRINITY_DN23308_c0_g1_i4	FSMCSP3	318	JAV45879.1 Chemosensory protein 2 [*Mythimna separata*]	132.1	8.00E-28	77.9	2
4	TRINITY_DN23308_c0_g2_i1	FSMCSP4	512	ABM67689.1 Chemosensory protein CSP2 [*Spodoptera exigua*]	200.7	3.00E-48	84.5	3288
5	TRINITY_DN23308_c0_g3_i1	FSMCSP5	600	ABM67689.1 Chemosensory protein CSP2 [*Spodoptera exigua*]	203.4	5.40E-49	82.7	5973
6	TRINITY_DN24471_c1_g3_i3	FSMCSP6	906	AAK53762.1 Chemosensory protein [*Helicoverpa armigera*]	217.6	4.20E-53	85.5	61490
7	TRINITY_DN24577_c3_g1_i1	FSMCSP7	840	ALJ30223.1 Putative chemosensory protein CSP12 [*Spodoptera litura*]	198	3.20E-47	88.5	36793
8	TRINITY_DN2461_c0_g1_i1	FSMCSP8	521	ALT31614.1 Chemosensory protein 32, partial [*Cnaphalocrocis medinalis*]	204.1	2.70E-49	94.2	22
9	TRINITY_DN24883_c0_g1_i3	FSMCSP9	1330	AGH20055.1 Chemosensory protein 17, partial [*Helicoverpa armigera*]	215.3	3.00E-52	90.7	47697
10	TRINITY_DN24883_c0_g1_i3	FSMCSP10	1330	AGH20055.1 Chemosensory protein 17, partial [*Helicoverpa armigera*]	215.3	3.00E-52	90.7	47697
11	TRINITY_DN25571_c0_g1_i1	FSMCSP11	1450	JAV45876.1 Chemosensory protein 5 [*Mythimna separata*]	184.1	8.20E-43	65.1	10413
12	TRINITY_DN25685_c1_g1_i1	FSMCSP12	1710	ALJ30215.1 Putative chemosensory protein CSP4 [*Spodoptera litura*]	347.8	5.00E-92	65.9	805

13	TRINITY_DN25796_c3_g3_i1	FSMCSP13	865	JAV45878.1 Chemosensory protein 3 [*Mythimna separata*]	204.1	4.50E-49	63.3	2163 6
14	TRINITY_DN25954_c2_g2_i1	FSMCSP14	503	ALT31609.1 Chemosensory protein 27, partial [*Cnaphalocrocis medinalis*]	191	2.30E-45	73.4	2795
15	TRINITY_DN25977_c6_g1_i3	FSMCSP15	622	ALJ30214.1 Putative chemosensory protein CSP3 [*Spodoptera litura*]	195.3	1.50E-46	73.2	394
16	TRINITY_DN8612_c0_g1_i1	FSMCSP16	1042	AGR39575.1 Chemosensory protein 5 [*Agrotis ipsilon*]	186	1.50E-43	85	62
17	TRINITY_DN18655_c0_g1_i1	FSMCSP17	816	AND82445.1 Chemosensory protein 3 [*Athetis dissimilis*]	216.1	1.10E-52	85.2	95

Note- Above some unigenes are coded by same protein id. but with different reading frames

Supplementary Table S5. Unigenes of candidate sensory neuron membrane proteins in *Eudocima materna* L. antennae transcriptome.

S. No	Transcript/ Unigene Id.	Gene name	Unigene length (bp)	BLASTx annotation	Score	*E*-value	Identity percent	Cou nt
1	TRINITY_DN24655_c4_g2_i4	FSMSNMP1	2131	AGN52676.1 Sensory neuron membrane protein 1 [*Spodoptera exigua*]	676.8	5.90E-191	59.7	239

2	TRINITY_DN25906_c0_g2_i1	FSMSNMP2	3217	Q9U1G3.1<br>Sensory neuron membrane protein 1; Short=SNMP1-HvirCAB65739.1 [*Heliothis virescens*]	737.3	5.50E-209	64.3	659<br>7
3	TRINITY_DN25906_c0_g2_i3	FSMSNMP3	3060	JAV45786.1<br>Sensory neuron membrane protein 1 [*Mythimna separata*]	769.2	1.30E-218	72.5	864<br>6
4	TRINITY_DN25931_c1_g2_i11	FSMSNMP4	2437	AGN48099.1<br>Sensory neuron membrane protein 2 [Spodoptera litura]	838.6	1.30E-239	74.5	717<br>72
5	TRINITY_DN25931_c1_g2_i9	FSMSNMP5	909	B2RFN2.1<br>Sensory neuron membrane protein 2; Short=HvirSNMP-2CAP19028.1 [*Heliothis virescens*]	276.6	7.60E-71	73.4	230<br>20

Note- Above some unigenes are coded by same protein id. but with different reading frames

Supplementary Table S6. Unigenes of candidate Antennal binding proteins in *Eudocima materna* L. antennae

S. No	Transcript/ Unigene Id.	Gene name	Unigene length (bp)	BLASTx annotation	Score	*E*-value	Identity percent	Count
1	TRINITY_DN13090_c0_g1_i1	FSMABP1	684	APD13867.1<br>Putative carboxylesterase CXE1 [*Spodoptera litura*]	246.9	4.80E-62	73	19
2	TRINITY_DN14063_c0_g1_i1	FSMABP2	388	AAY22446.1<br>Antennal aldehyde oxidase, partial [*Mamestra brassicae*]	224.2	1.90E-55	78.1	26
3	TRINITY_DN14671_c0_g1_i1	FSMABP3	413	ACV60247.1<br>Antennal esterase CXE20 [*Spodoptera littoralis*]	159.5	6.10E-36	55.6	17
4	TRINITY_DN18889_c0_g1_i1	FSMABP4	1610	KOB64960.1<br>Protein distal antenna [*Operophtera brumata*]	150.6	1.10E-32	70.7	131
5	TRINITY_DN20349_c0_g1_i1	FSMABP5	1815	AID54902.1	737.6	2.40E-209	64.7	631

				cytochrome P450 CYP9A14v2 [*Helicoverpa armigera*]				
6	TRINITY_DN22685_c0_g1_i1	FSMABP6	1686	AEJ38206.1<br>Antennal esterase CXE11 [*Spodoptera exigua*]	749.6	5.70E-213	65.7	157
7	TRINITY_DN23112_c0_g1_i1	FSMABP7	1583	ACV60246.1<br>Antennal esterase CXE19 [*Spodoptera littoralis*]	934.1	1.50E-268	83.3	146
8	TRINITY_DN23847_c5_g1_i1	FSMABP8	647	AII21997.1<br>Aldehyde oxidase AOX2 [*Sesamia inferens*]	121.3	2.90E-24	80.3	274
9	TRINITY_DN24224_c1_g1_i1	FSMABP9	1786	AKH15488.1<br>Cytochrome P450 [*Spodoptera litura*]	655.2	1.50E-184	58.1	313
10	TRINITY_DN24573_c3_g3_i1	FSMABP10	2159	AAR26518.1<br>Antennal cytochrome P450 CYP9 [*Mamestra brassicae*]	822.4	8.80E-235	76.1	1212
11	TRINITY_DN24573_c3_g5_i1	FSMABP11	2108	AAY21809.1<br>cytochrome P450 [*Helicoverpa armigera*]	758.4	1.50E-215	69.6	328
12	TRINITY_DN25128_c1_g3_i2	FSMABP12	2320	ACV60242.1<br>Antennal esterase CXE15 [*Spodoptera littoralis*]	634.8	2.80E-178	54.9	34523
13	TRINITY_DN25130_c3_g2_i2	FSMABP13	1688	NP_001116814.1<br>Alpha-esterase 40 precursor [*Bombyx mori*]	569.3	1.00E-158	55	279
14	TRINITY_DN25130_c3_g4_i1	FSMABP14	2172	ADR64698.1<br>Antennal esterase CXE18 [*Spodoptera litura*]	860.9	2.20E-246	75	301
15	TRINITY_DN25353_c7_g1_i2	FSMABP15	1868	AII21984.1<br>Odorant degrading enzyme CXE10 [*Sesamia inferens*]	702.6	8.80E-199	60.6	3991
16	TRINITY_DN25406_c0_g1_i1	FSMABP16	1640	ALT14583.1<br>Carboxylesterase [*Helicoverpa armigera*]	709.5	6.30E-201	61.2	696
17	TRINITY_DN25406_c1_g1_i1	FSMABP17	2181	ADF43458.1<br>Carboxyl/choline esterase CCE001b [*Helicoverpa armigera*]	609.8	9.00E-171	58	741
18	TRINITY_DN25409_c2_g1_i1	FSMABP18	750	XP_013141552.1<br>Esterase B1-like [*Papilio polytes*]	87.4	5.40E-14	64.8	749
19	TRINITY_DN25409_c2_g1_i2	FSMABP19	2006	AEJ38207.1<br>Antennal esterase CXE10 [*Spodoptera exigua*]	775	1.50E-220	65.6	3651
20	TRINITY_DN25409_c2_g1_i2	FSMABP20	2006	AEJ38207.1<br>Antennal esterase CXE10 [*Spodoptera exigua*]	775	1.50E-220	65.6	3651
21	TRINITY_DN25476_c0_g1_i1	FSMABP21	1420	AII21980.1<br>Odorant degrading enzyme CXE3 [*Sesamia inferens*]	654.4	2.10E-184	67.1	1709
22	TRINITY_DN25476_c0_g1_i2	FSMABP22	391	AKS40354.1<br>Antennal carboxylesterase 2, partial [*Chilo suppressalis*]	206.8	3.10E-50	83.8	36

23	TRINITY_DN25476_c0_g2_i1	FSMABP23	2172	ADR64703.1<br>Antennal esterase CXE20 [*Spodoptera exigua*]	737.3	3.70E-209	63.7	655
24	TRINITY_DN25491_c0_g2_i2	FSMABP24	642	ACV60244.1<br>Antennal esterase CXE17 [*Spodoptera littoralis*]	87	6.00E-14	61.9	73
25	TRINITY_DN25491_c0_g2_i5	FSMABP25	2053	ADR64700.1<br>Antennal esterase CXE17 [*Spodoptera exigua*]	780	4.80E-222	68.6	997
26	TRINITY_DN25586_c1_g2_i1	FSMABP26	434	AII21997.1<br>Aldehyde oxidase AOX2 [*Sesamia inferens*]	201.4	1.50E-48	62.7	15
27	TRINITY_DN25731_c0_g2_i2	FSMABP27	4573	ACV60236.1<br>Antennal esterase CXE9 [*Spodoptera littoralis*]	808.9	2.10E-230	68.2	1657
28	TRINITY_DN25731_c0_g2_i4	FSMABP28	3494	ACV60236.1<br>Antennal esterase CXE9 [*Spodoptera littoralis*]	808.9	1.60E-230	68.2	279
29	TRINITY_DN25731_c0_g2_i4	FSMABP29	3494	ACV60236.1<br>Antennal esterase CXE9 [*Spodoptera littoralis*]	808.9	1.60E-230	68.2	279
30	TRINITY_DN25783_c4_g1_i1	FSMABP30	1223	ADF43459.1<br>Carboxyl/choline esterase CCE001c [*Helicoverpa armigera*]	379	1.50E-101	66.4	168
31	TRINITY_DN25783_c4_g2_i1	FSMABP31	321	AEJ38205.1<br>Antennal esterase CXE14 [*Spodoptera exigua*]	170.2	2.70E-39	69.8	10
32	TRINITY_DN25783_c4_g3_i1	FSMABP 32	1771	AEJ38205.1<br>Antennal esterase CXE14 [*Spodoptera exigua*]	800.4	2.90E-228	68	2242
33	TRINITY_DN26025_c9_g2_i1	FSMABP 33	511	AKI87964.1<br>Odorant binding protein 3 [*Spodoptera litura*]	87.8	2.80E-14	80	44461
34	TRINITY_DN26025_c9_g5_i1	FSMABP34	364	AAP57463.1<br>Antennal binding protein X-1, partial [*Agrotis ipsilon*]	131.3	1.60E-27	79.5	76610
35	TRINITY_DN26076_c10_g2_i1	FSMABP35	2063	ALT14583.1<br>Carboxylesterase [*Helicoverpa armigera*]	737.6	2.70E-209	62.1	72448
36	TRINITY_DN31079_c0_g1_i1	FSMABP36	365	XP_014370995.1<br>F-BAR domain only protein 2 [*Papilio machaon*]	212.2	7.00E-52	89.3	15
37	TRINITY_DN32718_c0_g1_i1	APB37	343	AII21992.1<br>Odorant degrading enzyme CXE20 [*Sesamia inferens*]	180.6	2.10E-42	69.9	7
38	TRINITY_DN37783_c0_g1_i1	FSMABP38	792	AGS49137.1<br>carboxylesterase [*Agrotis ipsilon*]	412.1	1.00E-111	70	26
39	TRINITY_DN5331_c0_g1_i1	FSMABP39	901	AAF12781.1	464.5	1.90E-127	92.1	37

				Antennal specific membrane protein AMP [*Manduca sexta*]				
40	TRINITY_DN621_c0_g1_i1	FSMABP40	413	AEJ38205.1<br>Antennal esterase CXE14 [*Spodoptera exigua*]	235.7	6.70E-59	78.8	11
